# A STING signaling relay from tumor cells to macrophages mediates the improved efficacy of combination chemotherapy in pancreatic cancer

**DOI:** 10.1186/s12929-026-01226-1

**Published:** 2026-03-04

**Authors:** Honglu Ding, Yize Mao, Zehui Yao, Kaili Xing, Qiuxia Yang, Ruiqi Wang, Jun Wang, Yongxiang Liu, Hui Guo, Zining Wang, Xiaojuan Wang, Jinheng Wang, Jing Xue, Shengping Li, Xiaojun Xia

**Affiliations:** 1 Department of Pancreatobiliary Surgery, State Key Laboratory of Oncology in South China, Guangdong Provincial Clinical Research Center for Cancer, Sun Yat-Sen University Cancer Center, Guangzhou, 510060 China; 2 The Third Affiliated Hospital, Sun Yat-Sen University, Guangzhou, 510630 China; 3 Department of Medical Imaging, State Key Laboratory of Oncology in South China, Guangdong Provincial Clinical Research Center for Cancer, Sun Yat-Sen University Cancer Center, Guangzhou, 510060 China; 4 Guangzhou National Laboratory, Guangzhou International Bio-Island, Guangzhou, 510320 China; 5 State Key Laboratory of Oncology in South China, Guangdong Provincial Clinical Research Center for Cancer, Sun Yat-Sen University Cancer Center, Guangzhou, 510060 China; 6 The Affiliated Traditional Chinese Medicine Hospital, Guangzhou Medical University, Guangzhou, 510130 China; 7 State Key Laboratory of Systems Medicine for Cancer, Stem Cell Research Center, Renji Hospital, Shanghai Cancer Institute, Shanghai Jiaotong University School of Medicine, Shanghai, 200127 China; 8 Hainan Academy of Medical Sciences, Hainan Medical University, Haikou, 571199 China

**Keywords:** Pancreatic ductal adenocarcinoma, cGAS-STING pathway, Tumor microenvironment, Tumor-associated macrophages, Chemotherapy

## Abstract

**Background:**

The therapeutic efficacy of traditional chemotherapy on pancreatic ductal adenocarcinoma (PDAC) remains dismal. In this study, we investigated the efficacy of adding cisplatin to the standard first-line gemcitabine plus nab-paclitaxel (AG) regimen (referred to as AGP) for PDAC treatment, and elucidated the underlying mechanisms, particularly the role of the cGAS-STING pathway in mediating chemotherapy-induced antitumor immunity in PDAC.

**Methods:**

We first reported the therapeutic efficacy of an AGP regimen in patients with PDAC through a clinical retrospective analysis. Next, we mimicked the enhanced efficacy of the AGP regimen in both subcutaneous and orthotopic PDAC mouse models. Comprehensive immune profiling was performed using mass cytometry, flow cytometry, multiplex immunofluorescence, and RNA sequencing to characterize changes in immune cell populations and phenotypes. The functional significance of the cGAS-STING pathway was investigated through genetic ablation of tumor cells and macrophages. Tumor–macrophage interactions were further explored via co-culture assays. Clinical relevance was assessed through a retrospective analysis of cohorts of patients with PDAC and immunohistochemical evaluation of STING expression in tumor tissues.

**Results:**

The AGP regimen confers promising potential to AG regimen in patients with PDAC as well as in PDAC mouse models. Mechanistically, cisplatin-induced DNA damage in tumor cells activated the tumor-intrinsic cGAS-STING pathway, which facilitated the recruitment and activation of CD8^+^ T cells. Furthermore, phagocytosis of tumor-derived damage-associated molecular patterns by tumor-associated macrophages (TAMs) triggered the activation of cGAS-STING signaling and promoted M1 polarization of TAMs without obvious macrophage cell death. Such “STING signaling relay” between tumor cells and TAMs reprogrammed the tumor microenvironment and facilitated chemotherapy efficacy. Clinically, high STING expression in PDAC tissues was associated with increased infiltration of cytotoxic T cells and M1-like macrophages, and was identified as an independent predictor of improved patient prognosis.

**Conclusions:**

This study reports AGP regimen as a promising therapeutic modality for PDAC, and provides a detailed mechanism by which a STING-mediated signaling relay from PDAC tumor cells to TAMs boost antitumor immunity and contribute to AGP chemotherapy efficacy. Furthermore, STING expression in tumor tissues correlated with improved prognosis, highlighting its potential as a predictive biomarker and promising therapeutic target.

**Graphical Abstract:**

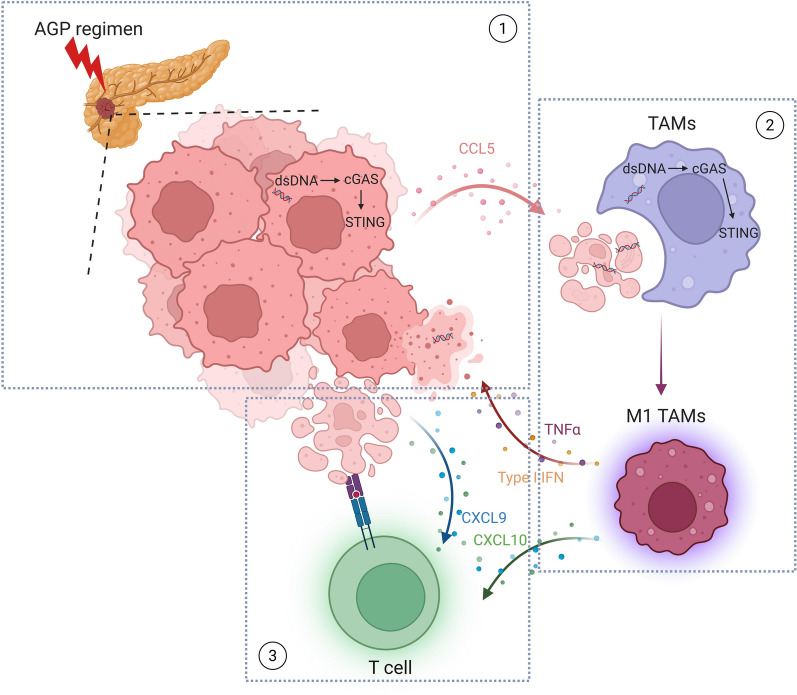

**Supplementary Information:**

The online version contains supplementary material available at 10.1186/s12929-026-01226-1.

## Introduction

Pancreatic ductal adenocarcinoma (PDAC) is one of the most aggressive malignancies, with a 5-year overall survival (OS) rate of only 13% [1]. Its incidence is steadily increasing, and it is projected to become the second leading cause of cancer-related deaths by 2030 [2]. Approximately 80% of patients are diagnosed at an advanced stage, which precludes surgical resection [3]. Hence, chemotherapy remains the mainstay treatment for advanced pancreatic cancer (APC). However, despite incremental advances, current first-line regimens such as FOLFIRINOX and the combination of gemcitabine and albumin-bound paclitaxel (AG regimen) provide only limited improvements in OS and quality of life [4, 5], underscoring the urgent need for more effective therapeutic strategies.

The limited efficacy of chemotherapy in PDAC is multifactorial. Both intrinsic and acquired resistance to cytotoxic agents, combined with a dense desmoplastic stroma and profoundly immunosuppressive tumor microenvironment (TME), severely restrict drug delivery and antitumor immune responses [6–9]. The TME in PDAC is characterized by low effector T cell infiltration and a predominance of immunosuppressive populations, including regulatory T cells (Tregs), myeloid-derived suppressor cells (MDSCs), and M2-polarized tumor-associated macrophages (TAMs) [10]. These features diminish the efficacy of chemotherapy and contribute to the failure of immunotherapeutic approaches, highlighting the need for innovative strategies to overcome these barriers. Recent evidence suggests that reprogramming the TME to an immune-active state significantly enhances therapeutic responses [11].

Advances in tumor immunology have underscored the pivotal role of innate immune-sensing pathways in shaping antitumor immunity. Among these, the cyclic guanosine monophosphate-AMP synthase stimulator of interferon genes (cGAS-STING) pathway has emerged as a central mediator of both cancer cell-intrinsic and immune cell-mediated responses to DNA damage [12, 13]. Activation of the cGAS-STING pathway leads to the production of type I interferons and pro-inflammatory cytokines, which enhance antigen presentation, promote recruitment of cytotoxic T cells, and reprogram the TME from an immune-cold to an immune-active state [14, 15]. Intratumoral injection of STING agonists demonstrated significant immune-activating and tumor-inhibiting effect on PDAC mouse models, but so far there is still no clinically approved STING agonist for patient treatment [16, 17]. Notably, certain chemotherapeutic agents, particularly platinum-based compounds such as cisplatin, are potent inducers of DNA damage and have been shown to activate the cGAS-STING pathway [18]. However, the potential of leveraging this immunogenic mechanism to improve chemotherapeutic efficacy in PDAC remains underexplored.

This study investigated the combination of cisplatin with an AG regimen (AGP regimen) for PDAC treatment for enhancing chemotherapeutic efficacy through TME reprogramming mediated by cGAS-STING pathway activation. We systematically evaluated the therapeutic efficacy and underlying mechanisms of this regimen using clinical samples and preclinical models. Our findings demonstrated that the AGP regimen reshapes the tumor immune microenvironment (TIME), enhances antitumor immunity, and improves chemotherapy efficacy by inducing a relay in the cGAS-STING signaling cascade between tumor cells and TAMs. These results provide a promising chemotherapy strategy for PDAC and highlight the critical role of the cGAS-STING pathway in shaping the TIME and mediating the therapeutic efficacy of the AGP regimen.

## Materials and methods

### Patients and tissue samples

A retrospective analysis was conducted involving 108 patients with APC who received either the AG or AGP therapeutic regimen at Sun Yat-sen University Cancer Center (SYSUCC) between January 2019 and December 2023. Eligible patients were required to meet the following criteria: (1) histologically or cytologically confirmed pancreatic cancer; (2) radiographic evidence of APC (Stage III and IV); (3) absence of concurrent radiotherapy; (4) no other synchronous primary malignancies; and (5) availability of baseline and follow-up imaging data for response evaluation. Additionally, 56 patients with PDAC who underwent surgical resection or irreversible electroporation ablation at SYSUCC between January 2018 and December 2021 were included for tumor tissue collection and analysis. The tissue samples were formalin-fixed and paraffin-embedded. This retrospective study was approved by the Research Ethics Committee of SYSUCC. Patient demographic data, including age, sex, Eastern Cooperative Oncology Group (ECOG) performance status, primary tumor location, primary tumor size, presence of distant metastasis, presence of metastases within multiple different organs, and the treatment process, were retrieved from electronic medical records.

### Evaluation of treatment response, endpoints, and follow-up

Treatment response was assessed by an expert radiologist 2–3 months after chemotherapy initiation, using RECIST 1.1 criteria [19]. Overall survival (OS, in months) was defined from the date of initiation of chemotherapy to the date of death or the date of last follow-up. Progression-free survival (PFS, in months) was defined from the date of initiation of chemotherapy to the date of first imaging assessment as disease progression or the date of death. Follow-up continued until December 31, 2023.

### Cell culture

All cell lines were routinely tested as mycoplasma-negative and were not passaged for more than 3 months. PDAC cells were originally isolated from primary pancreatic tumors of KPC mice with a mixed 129/C57BL/6 genetic background. Genotyping was conducted using PCR with primers targeting mutant *Kras* (*Kras*-F:5′-GGGTAGGTGTTGGGATAGCTG-3′,*Kras*-R:5′TCCGAATTCAGTGACTACAGATGACAGAG-3′) and *p53*^*R172H*^ recombination (*p53*-Re-F: 5′-AGCCTGCCTAGCTTCCTCAGG-3′, *p53*-Re-R: 5′-CTTGGAGACATAGCCACACTG-3′) [20]. KPC-1199 cells were derived from the primary pancreatic tumor of a KPC mouse with a C57BL/6 background [20]. Cells were cultured in Dulbecco’s modified Eagle’s medium or RPMI1640 (Invitrogen) medium supplemented with 10% fetal bovine serum (FBS) and 1% penicillin–streptomycin at 37 °C with 5% CO_2_. Drug concentrations were as follows: gemcitabine (10 μM), nab-paclitaxel (10 μM), cisplatin (5 μM), the specific cGAS inhibitor RU.521 (2262452-06, TargetMol, 10 μM), and the cytoskeletal inhibitor Cytochalasin D (S8184, Selleck, 10 μM).

### Animal models

Female C57BL/6 J mice (6–8 weeks-old) were obtained from Vital River Laboratory (Beijing, China). *Sting*^*flox/flox*^ mice were purchased from Shanghai Model Organism Inc. and were crossed with *LysM-Cre* mice to generate macrophage-specific *Sting* knockout mice. *Cgas*-KO mice were purchased from GemPharmatech (Nanjing, China). All animals were maintained under specific pathogen-free conditions, and all procedures were approved by the Institutional Animal Care and Use Committee of Sun Yat-sen University.

### Tumor implantation and treatment

For subcutaneous models, 1 × 10^6^ PDAC cells were suspended in 100 μL of phosphate-buffered saline (PBS) and injected into mice. For orthotopic models, 1 × 10^6^ PDAC cells were mixed with 50 μL of PBS and Matrigel (1:1) and injected into the pancreas. In the KPC-1199 model, 3 × 10^6^ cells were suspended in a 100 μL mixture of PBS and Matrigel (4:1) and injected subcutaneously. The AGP regimen consisted of gemcitabine (50 mg/kg), albumin-bound paclitaxel (5 mg/kg), and cisplatin (5 mg/kg) administered twice weekly for 2 weeks via intraperitoneal injection [21–23].

### Generation of bone marrow-derived macrophages (BMDMs)

Bone marrow cells were isolated from femurs and tibias of B6 mice, erythrocytes lysed, and cells were cultured in RPMI 1640 with 10% FBS, 1% antibiotics, 10% conditioned medium from L929 cells, 55 mM β-mercaptoethanol, 1 mM sodium pyruvate, and 2 mM GlutaMax.

### LacZ activity measurement

LacZ activity was measured as previously described [24]. B3Z T cells were lysed using 50 mL of LacZ lysis buffer, subjected to freeze–thaw cycles, then mixed with 50 mL of PBS containing 0.5% bovine serum albumin and 100 mL of substrate solution (1 mg/mL chlorophenol red β-D-galactopyranoside) dissolved in β-galactosidase buffer. Subsequently, the samples were incubated at 37 ℃ for 5–10 h for color development, and the absorbance was measured at 590 nm.

### T cell activation assay

PDAC-OVA cells were co-cultured with bone marrow-derived dendritic cells (BMDCs) or BMDMs and B3Z T cells at a ratio of 1:1:5 at the specified time points. LacZ activity and interleukin (IL)−2 secretion were measured using a LacZ assay and enzyme-linked immunosorbent assay (ELISA), respectively (eBioscience, 88–7024-88).

### CRISPR/Cas9‐mediated gene knockout

*Sting*-deficient cells were generated using CRISPR/Cas9 technology [20].The sgRNA sequences were designed using the Optimized CRISPR Design tool (http://chopchop.cbu.uib.no/). The specific guide sequence for *Sting* was 5′-GTACCTTGGTAGACAATGAGG-3′, and the sgRNA was cloned into the LentiCRISPR v2 vector. Following transient transfection with the lenti-CRISPR v2 vector, cells were selected using puromycin. The knockout efficiency was confirmed via western blot analysis.

### Flow cytometry

Single-cell suspensions were prepared from tissues or cultured cells. For cell surface marker analysis, antibodies were incubated according to the manufacturer’s instructions. For intracellular marker analysis, after sufficient fixation and permeabilization, cells were incubated with intracellularly stained antibodies. Flow cytometry analysis was performed using a BD LSR Fortessa X-20 Cell Analyzer, and sorting was performed using a BD FACSAria III Cell Sorter. Data were processed using FlowJo software. The flow cytometry antibodies used were as follows: Brilliant Violet 421^™^ anti-mouse CD45 (103134, Biolegend), APC anti-mouse CD3 (100236, Biolegend), PE/Cyanine7 anti-mouse CD4 (100422, Biolegend), FITC anti-mouse CD8a (100705, Biolegend), PerCP/Cyanine5.5 anti-mouse/human CD11b (101228, Biolegend), FITC anti-mouse F4/80 (123108, Biolegend), Brilliant Violet 421^™^ anti-mouse H-2 Kb (116525, Biolegend), PE anti-mouse CD274 (B7-H1, PD-L1) (124308, Biolegend), FITC anti-mouse CD80 (104706, Biolegend), and Alexa Fluor^®^ 647 anti-mouse CD206 (MMR) (141712, Biolegend).

### Mass cytometry (CyTOF)

A 42-marker metal-conjugated antibody panel was used to profile immune cell lineages and functional markers (Table S1). Antibodies were either commercially preconjugated (Fluidigm) or labeled in-house with heavy metal isotopes using a Maxpar X8 Antibody Labeling Kit (Fluidigm). Tumor tissues were processed into single-cell suspensions. Cells were incubated with cisplatin (5 μM) for live or dead discrimination, and marked with a barcode after fixation. Surface staining was performed using a pre-titrated antibody cocktail. For intracellular markers, the cells were fixed or permeabilized with FOXP3/Transcription Factor Buffer (Thermo Fisher Scientific) and stained with intracellular antibodies. Finally, the cells were fixed in 2% paraformaldehyde and incubated overnight with an iridium intercalator (62.5 nM) for DNA labeling. Samples were analyzed using a CyTOF2 mass cytometer (Fluidigm). Raw FCS files were normalized using bead-based algorithms (https://premium.cytobank.cn/).

### Immunofluorescence

The cells were fixed, permeabilized, blocked, and incubated with primary and secondary antibodies. Nuclei were stained with DAPI (Sigma). The images were acquired using a Zeiss LSM880 confocal microscope. The antibodies used were as follows: γ-H2AX (9718, CST), STING (13647, CST), dsDNA (MAB 1293, Milipore), F4/80 (30325, CST), Goat anti-rabbit IgG H&L (Alexa Fluor^®^ 488) (Abcam, ab150077), and Goat anti-rabbit IgG H&L (Alexa Fluor^®^ 555) (Abcam, ab150078).

### Multiplexed immunofluorescence staining (mIF) and analysis

Tissue sections were deparaffinized in xylene and rehydrated in a series of ethanol baths (100%, 95%, 90%, 80%, and 70%). Multiplexed staining was conducted using the Tyramide Signal Amplification kit (TissueGnostics, TGFP7100) according to the manufacturer’s protocol. The antibodies used were as follows: PanCK (19003-1-ap, Proteintech), STING (13647, CST), CD68 (ab213363, Abcam) and F4/80 (70076, CST). Nuclei were counterstained with DAPI. Images were analyzed using the AP-TIME image analysis software (3D Medicines, Inc.) [25] to quantify cell densities and proportions. Spatial organization was evaluated by calculating the normalized nearest-neighbor distances between PAN-CK^+^ tumor cells and CD68^+^ macrophages.

### Immunohistochemistry

Tissue sections were deparaffinized, rehydrated, repaired with ethylene-diamine-tetraacetic acid buffer, and incubated with primary antibodies. Signals were detected using a chromogenic substrate (Dako, K5007) and counterstained with hematoxylin. Images were scanned and saved using an automatic slide scanner. Images were analyzed using the HALO image analysis platform (Indica Labs, USA). The AI classifier of HALO platform was trained by annotating representative regions containing moderately stained nuclei (DAPI, blue) and target protein signals (DAB, brown). Based on optical density (OD) thresholds, the system automatically classified each detected cell into one of four categories: negative, weak positive, moderate positive, or strong positive. The platform analyzed the entire tissue region and calculated the percentage area of each intensity category relative to the total scanned area. Quantitative data exported from HALO were integrated with patient survival times. X-tile software, a validated tool for biomarker cut-off optimization [26], was used to identify the STING expression threshold that most significantly stratified patients by survival outcome. Based on this objective, prognosis-based cut-off, patients were categorized into “STING-High” and “STING-Low” groups for subsequent analyses.The antibodies used were as follows: STING (13647, CST), CD86 (91882, CST), CD206 (91992, CST), and CD8a (85336, CST).

### ELISA

Levels of IL-2 (88–7024, Invitrogen), IL-6 (88–7064, Invitrogen), CCL5 (88–56009, Invitrogen), CXCL10 (BMS6018, Invitrogen), tumor necrosis factor-alpha (TNF-α; 88–7324, Invitrogen), and interferon beta (IFN-β; A47435, Invitrogen) in culture supernatants were measured using commercial ELISA kits according to the manufacturer’s instructions.

### Western blot

Protein extraction, SDS-PAGE, and western blotting were performed as previously described [27]. The antibodies used were as follows: β-actin (sc-8432, Santa Cruz), cGAS (31659, CST), STING (13647, CST), p-STING (50907, CST), IRF3 (4302, CST), p-IRF3 (29047, CST), TBK1 (3504, CST), p-TBK1 (5483, CST), PD-L1 (13684, CST), and iNOS (13120, CST).

### Real-time PCR

Total RNA was extracted from the cells using TRIzol reagent (Invitrogen) following the manufacturer's guidelines. RNA was reverse transcribed using the Primer Script RT Reagent Kit. Real-time PCR was conducted using the SYBR Premix kit (Genstar) and analyzed using a Bio-Rad detection system. Gene expression was normalized to actin using the ΔΔCT method. Primer sequences are provided in the Supplementary Information (Table S2).

### Bulk RNA sequencing

Total RNA was extracted from the cells using TRIzol reagent (Invitrogen) following the manufacturer's guidelines. Total RNA was submitted to Lianchuan Biotechnology Co. (Guangzhou, China) for RNA-sequencing. The specific analysis process was performed as previously described [28].

### TCGA cohort analyses

We retrieved pancreatic cancer transcriptomic data from the TCGA-PAAD project and included 139 samples histologically confirmed as invasive ductal carcinoma (not otherwise specified) for downstream analysis [29]. Gene expression was quantified in transcripts per million (TPM) and log2-transformed to stabilize variance for correlation analyses. Tumor microenvironment immune cell composition was deconvoluted using quanTIseq, yielding relative infiltration scores for 10 core immune cell types. Associations between STING expression (continuous) and each immune score were evaluated using Spearman correlation, with results visualized via ggplot2. All immune deconvolution and microenvironment analyses were streamlined using the IOBR framework, which facilitated batch processing and integrated visualization through a unified interface.

### Statistical analysis

All experiments were performed in at least three biological replicates. Data were analyzed using GraphPad Prism software. Two-group comparisons were performed using a two-tailed unpaired Student’s t-test, and multiple groups were compared using one-way or two-way analysis of variance with Bonferroni’s post-hoc test. Survival was analyzed using Kaplan–Meier and log-rank tests. Cox proportional hazards models were used for univariate and multivariate analyses. Statistical significance was set at p < 0.05.

## Results

### Clinical response to the AGP regimen

**Fig. 1:**
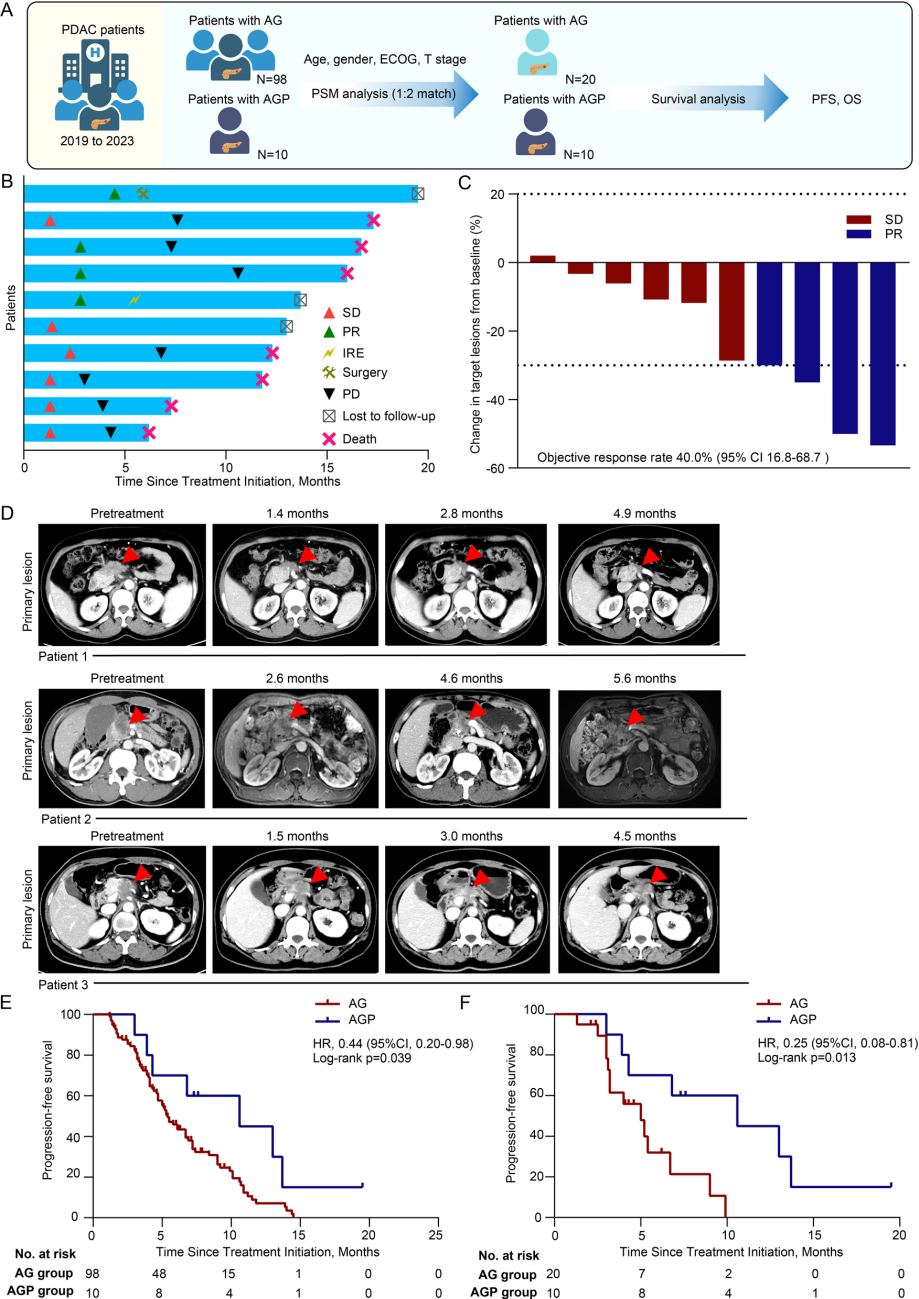
Clinical response to the AGP regimen. **A** Flowchart illustrating the process of propensity score matching for clinical cohort selection; **B** Swimmer plot depicting individual treatment durations, response events, and survival status for patients in the AGP regimen cohort; **C** Waterfall plot showing the maximum percentage change in target lesion size from baseline for each patient; the majority of AGP regimen-treated patients exhibited tumor shrinkage; **D** Representative imaging data demonstrating tumor shrinkage before and after AGP regimen treatment; red arrows indicate the primary lesion; **E**, **F** Kaplan–Meier survival curves showing significantly improved PFS in the AGP regimen group compared to the AG regimen group, both in unmatched and matched cohorts

We conducted a retrospective clinical study to analyze the efficacy of the AGP regimen for PDAC treatment. Overall, 108 patients with PDAC were included in this study; of these, 98 received the AG regimen and 10 received the AGP regimen. Propensity score matching was performed at a 1: 2 ratio to balance baseline characteristics, resulting in 10 matched pairs for the AGP group and 20 matched pairs for the AG group. These pairs were then analyzed for survival outcomes and treatment response (Fig. 1A). Patient characteristics before and after matching are summarized in Table 1. The baseline characteristics of patients in the AGP group are detailed in Table 2. The balance diagnostics results are provided in Figure S1A, indicating a good balance of baseline characteristics. Among the 10 patients treated with the AGP regimen, the objective response rate (ORR) was 40.0% (95% confidence intervals [CI] 16.8–68.7), with partial response (PR) observed in four patients and stable disease (SD) in the remaining patients (Fig. 1C). Individual treatment durations, response events, and survival status are illustrated in a swimmer plot (Fig. 1B). The waterfall plot shows the maximum tumor shrinkage from baseline in target lesions, with nine of the ten patients exhibiting tumor reduction, confirming the clinical benefit observed in most of the AGP regimen-treated individuals (Fig. 1C).

**Table 1:** Comparison of the baseline demographic and disease characteristics

	Before matching			After matching		
	AG (n = 98)	AGP (n = 10)	P	AG (n = 20)	AGP (n = 10)	P
Age			0.166			1
< 65	65 (66.3)	9 (90.0)		18 (90.0)	9 (90.0)	
≥ 65	33 (33.7)	1 (10.0)		2 (10.0)	1 (10.0)	
Gender			1			1
Female	45 (45.9)	4 (40.0)		8 (40.0)	4 (40.0)	
Male	53 (54.1)	6 (60.0)		12 (60.0)	6 (60.0)	
ECOG			0.001*			1
0	62 (63.3)	1 (10.0)		2 (10.0)	1 (10.0)	
1	30 (30.6)	9 (90.0)		18 (90.0)	9 (90.0)	
2	6 (6.1)	0 (0)		0 (0)	0 (0)	
Stage			0.478			1
LAPC	28 (28.6)	4 (40.0)		8 (40.0)	4 (40.0)	
mPC	70 (71.4)	6 (60.0)		12 (60.0)	6 (60.0)	

*AG* gemcitabine plus nab-paclitaxel, *AGP* gemcitabine plus nab-paclitaxel plus cisplatin, *ECOG-PS* Eastern Cooperative Oncology Group performance status, *LAPC* locally advanced pancreatic cancer, *mPC* metastatic pancreatic cancer

^*^indicates statistical significance

**Table 2:** Patient and tumor characteristics of AGP group (n = 10)

Variables	n (%)
Age (years)	
< 65	9 (90.0)
≥ 65	1 (10.0)
Gender	
Female	4 (40.0)
Male	6 (60.0)
ECOG	
0	1 (10.0)
1	9 (90.0)
2	0 (0)
BMI (kg/m^2^)	
< 24	9 (90.0)
> 24	1 (10.0)
Tumor size (cm)	
≤ 4	4 (40.0)
> 4	6 (60.0)
Tumor site	
Head/Neck	4 (40.0)
Body/Tail	6 (60.0)
Stage	
III	4 (40.0)
IV	6 (60.0)
CA19-9 (U/ml)	
≤ 35	2 (20.0)
> 35	8 (80.0)

Three patients with locally APC achieved sustained tumor shrinkage and PR over time (Fig. 1D). Patient 1 was initially assessed for SD at 1.4 months, achieved PR at 2.8 months, and maintained this response for 4.9 months, after which irreversible electroporation ablation (IRE) was performed. Patient 2 was assessed as having SD at 2.6 months, achieved PR at 4.6 months, maintained a response until 5.6 months, and subsequently underwent the Whipple procedure. Patient 3 achieved PR at 3.0 months and continued to respond in subsequent assessments, indicating the durable efficacy of the AGP regimen. The red arrows in each scan indicate the primary lesion, highlighting tumor shrinkage over time.

Unmatched survival analysis revealed that the AGP regimen was associated with significantly improved PFS compared with that of the AG regimen (hazard ratio [HR] = 0.44, 95% CI 0.20–0.98; p = 0.039; Fig. 1E). In the matched cohort, the AGP regimen continued to demonstrate a PFS advantage (HR = 0.25, 95% CI 0.08–0.81; p = 0.013; Fig. 1F). However, no significant difference in OS was observed between the two groups in either the unmatched cohort (HR = 0.80, 95% CI 0.36–1.75; p = 0.57; Figure S1B) or the matched cohort (HR = 1.12, 95% CI 0.42–2.99; p = 0.81; Figure S1C). The incidence of grade ≥ 3 adverse events is summarized in Table 3. Aside from a more pronounced decrease in platelet counts in the AGP group, the incidence of other adverse events was comparable between the two treatment groups.

**Table 3:** Grade ≥ 3 treatment adverse events

Adverse events	AG (n = 98)	AGP (n = 10)	P
Anemia	16 (16.3)	3 (30.0)	0.376
Leukopenia	28 (28.6)	3 (30.0)	1
Thrombocytopenia	18 (18.4)	5 (50.0)	0.034*
Fatigue	9 (9.2)	1 (10.0)	1
Nausea and vomiting	8 (8.2)	1 (10.0)	1
Fever	8 (8.2)	1 (10.0)	1
Pain	7 (7.1)	1 (10.0)	0.553
Peripheral neuropathy	12 (12.2)	1 (10.0)	1

^*^indicates statistical significance

Collectively, the AGP regimen confers promising potential in patients with PDAC, with a notable ORR and significant tumor shrinkage in most treated individuals. Representative cases showed sustained partial responses, highlighting its potential for subsequent curative interventions such as surgery or ablation. Survival analysis further confirmed a trend towards the PFS benefit of the AGP regimen over the AG regimen in both unmatched and matched cohorts. However, despite our efforts to expand it, the sample size of the AGP group remains limited. Therefore, these clinical findings should be interpreted as hypothesis-generating. They underscore the potential of this combinatorial approach but necessitate confirmation in larger, prospective studies to definitively establish its clinical benefit.

### AGP regimen demonstrates superior antitumor efficacy in mouse pancreatic cancer models

**Fig. 2:**
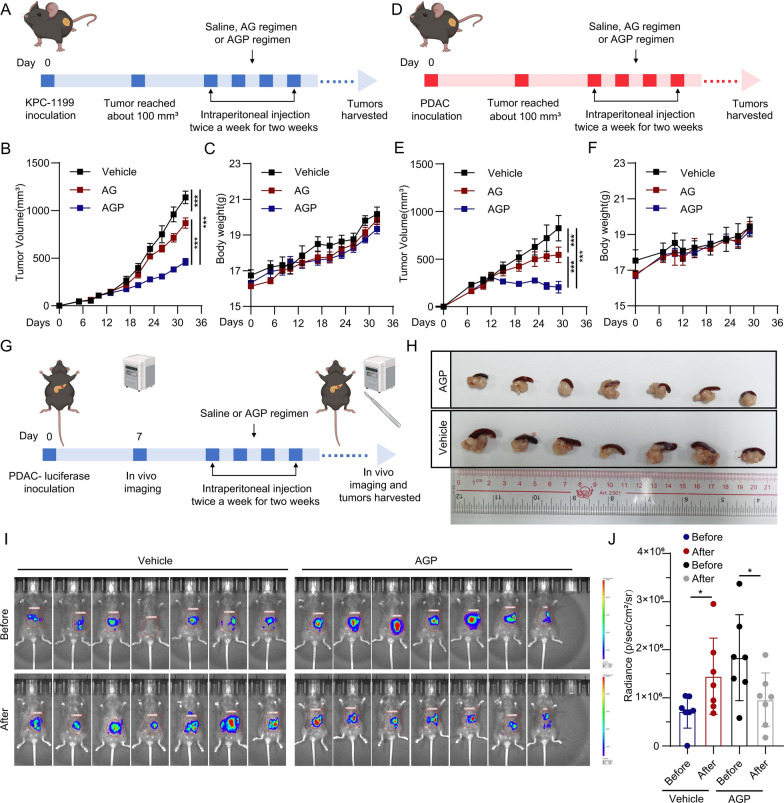
Therapeutic efficacy of the AGP regimen in mouse pancreatic cancer models. **A**, **D** Schematic diagram of subcutaneous tumor model for pancreatic cancer; **B**, **C** The curves of tumor growth and weight change of mice in subcutaneous tumor model of KPC-1199 cells (mean ± SEM; ***p < 0.001); **E**, **F** The curves of tumor growth and weight change of mice in subcutaneous tumor model of PDAC cells (mean ± SEM; ***p < 0.001); **G** Schematic diagram of orthotopic tumor model established with PDAC-luciferase cells; **H** Representative macroscopic images of orthotopic tumors from each treatment group; **I**, **J** Quantitative analysis of bioluminescence intensity in orthotopic tumors (radiant efficiency scale: 1 × 10^6^–4 × 10^6^ p/s/cm^2^/sr; *p < 0.05)

Considering the promising efficacy of the AGP regimen for PDAC, we assessed its therapeutic efficacy in mouse models of PDAC. First, we established subcutaneous PDAC tumor models in C57BL/6 mice using KPC-1199 and PDAC cells (Fig. 2A, D). Compared with the untreated control group, the AGP regimen significantly reduced tumor volume and outperformed the AG regimen (Fig. 2B, E; Figure S2A). Notably, no significant body weight fluctuations were observed between the treatment groups (Fig. 2C, F), suggesting favorable systemic tolerance.

Further, we assessed the therapeutic efficacy of the AGP regimen in an orthotopic pancreatic cancer model using PDAC-luciferase cells, facilitating the observation of dynamic tumor growth (Fig. 2G). Consistently, the AGP regimen effectively inhibited orthotopic tumor growth (Fig. 2H–J). Hematoxylin and eosin staining revealed extensive tumor necrosis in the AGP regimen group, whereas vital organs maintained normal histological architecture (Figure S2B, C). Hematology and serum biochemistry analyses revealed that the AGP regimen induced leukopenia, indicative of bone marrow suppression. Notably, the severity of this hematological toxicity was comparable to that observed with the AG regimen. Furthermore, AGP treatment significantly elevated serum alanine aminotransferase (ALT) levels, suggesting hepatocyte injury, whereas parameters for renal function and myocardial injury remained within normal ranges (Figure S2D, E). Collectively, these results demonstrate that the AGP regimen exhibits superior antitumor efficacy over the AG regimen in preclinical pancreatic cancer models, effectively inducing tumor regression with a comparable systemic safety profile, thereby underscoring its clinical potential.

### The AGP regimen remodels the pancreatic tumor immune microenvironment

**Fig. 3:**
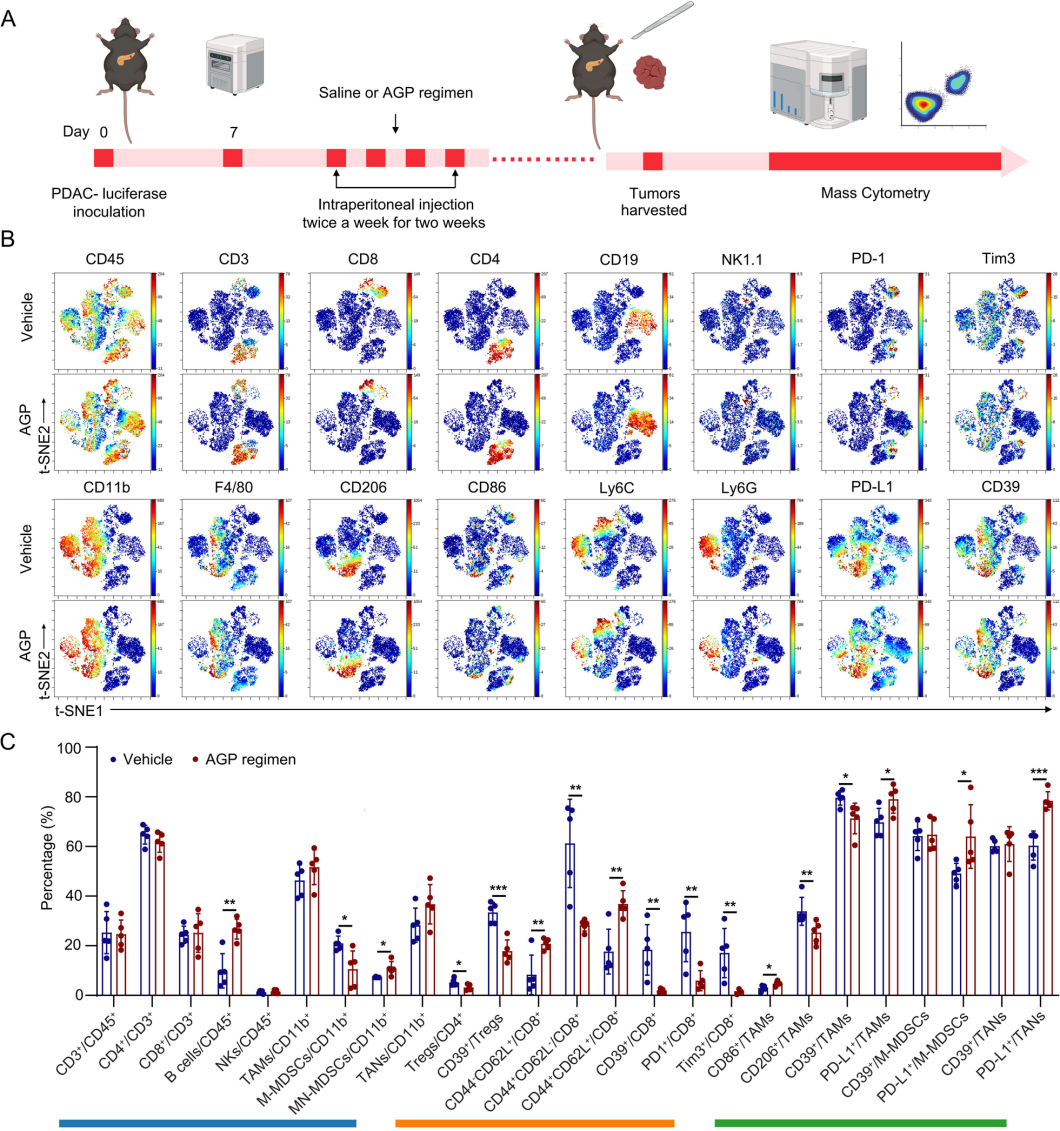
The AGP regimen remodels pancreatic tumor immune microenvironment. **A** Orthotopic tumors (n = 5/group) were dissociated and analyzed by CyTOF using a 42-marker panel; **B** viSNE maps colored by major immune lineages (t-SNE1 vs t-SNE2); **C** Quantitative analysis of immune cell infiltration and functional marker expression in the control group and AGP regimen group. The blue line represents changes of the overall cellular level, the orange line represents changes of the lymphocyte level, and the green line represents changes of the myeloid cell level (*p < 0.05, **p < 0.01, ***p < 0.001)

Considering that chemotherapy stimulates the TIME in addition to killing tumor cells [30], we assessed tumor immune infiltration after AGP treatment. Flow cytometric analysis of subcutaneous tumor tissues revealed increased infiltration of CD8^+^ T cells and elevated levels of TAMs following AGP treatment (Figure S3A, B). To assess the changes in the TME more comprehensively, we performed mass cytometry on orthotopic tumors from the control and AGP groups (Fig. 3A).

Using a 42-marker panel (Figure S3C), we constructed a high-dimensional profile of the immune landscape of PDAC tumors. ViSNE analysis enabled the qualitative characterization of the major immune populations and their phenotypic changes (Fig. 3B). Quantitative analysis (Fig. 3C) revealed that the AGP regimen increased B cell infiltration and significantly reduced the infiltration of M-MDSCs compared with that of the control group. Although infiltration of TAMs and tumor-associated neutrophils (TANs) increased, these changes were not significant. Further analysis revealed a significant reduction in Tregs and marked increase in naïve and memory CD8^+^ T cells. The expression of immunosuppressive molecules, including PD-1, Tim-3, and CD39, in CD8^+^ T cells was significantly downregulated in the AGP treatment group. Within the myeloid compartment, M1 TAMs increased prominently, whereas M2 TAMs and CD39 expression decreased. In contrast, PD-L1 expression was significantly elevated in TAMs, TANs, and M-MDSCs. Collectively, the AGP regimen reprogrammed the pancreatic TME toward an antitumor state by enhancing cytotoxic lymphocyte activity, alleviating immunosuppressive populations, and promoting proinflammatory myeloid polarization.

### AGP regimen potently activates the cGAS-STING pathway for superior antitumor efficacy

**Fig. 4:**
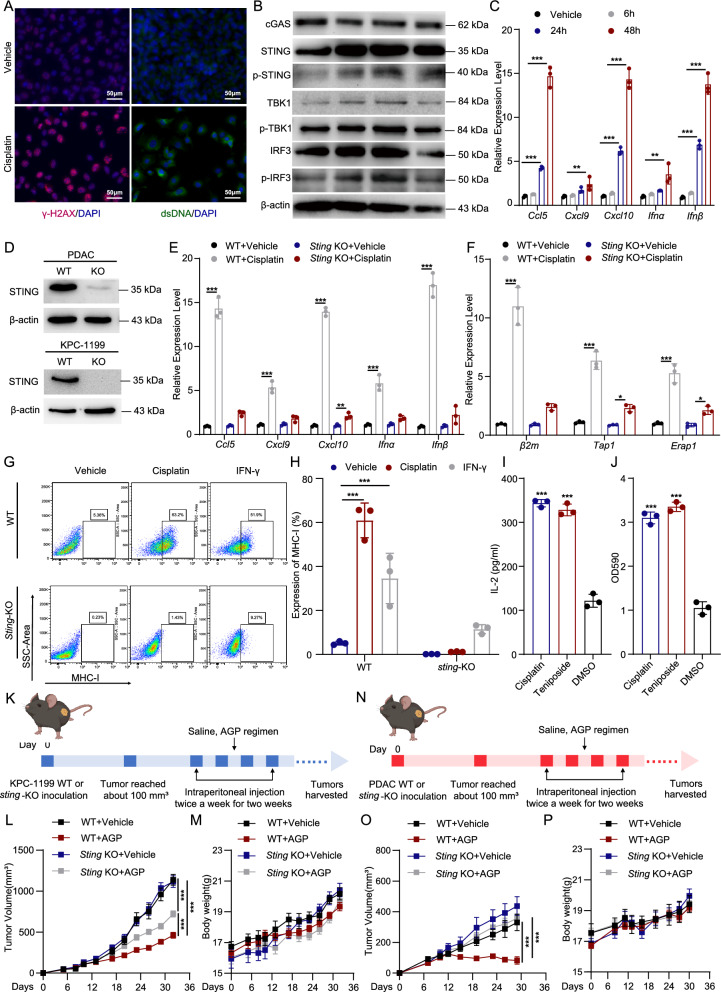
The AGP regimen activates cGAS-STING pathway for superior antitumor efficacy. **A** Immunofluorescence analysis of γ-H2AX (red) and dsDNA (green) in PDAC cells treated with cisplatin for 24 h; Nuclei stained with DAPI (blue); Scale bars, 50 µm; **B** Western blot analysis of cGAS-STING pathway-related proteins in cisplatin-treated PDAC cells; **C** qPCR analysis of type I interferon-related genes (*Ifnα*, *Ifnβ*) and chemokine genes (*Ccl5*, *Cxcl9*, *Cxcl10*) in PDAC cells treated with cisplatin for different durations (**p < 0.01, ***p < 0.001); **D** Validation of STING knockout in PDAC and KPC-1199 cells by Western blot; **E**, **F** qPCR analysis of type I interferon-related genes and MHC-I-related genes (*β2m*, *Tap1*, *Erap1*) in cisplatin-treated WT and *Sting*-KO cells (*p < 0.05, ***p < 0.001); **G**, **H** Flow cytometry analysis of MHC-I expression on WT and *Sting*-KO cells treated with cisplatin for 48 h. IFN-γ stimulation as a positive control (***p < 0.001); **I** LacZ activity assay in a co-culture system of PDAC-OVA cells, BMDCs, and B3Z T cells treated with cisplatin, teniposide and DMSO as positive and negative controls (**p < 0.01, ***p < 0.001); **J** ELISA quantification of IL-2 in the co-culture supernatant (**p < 0.01, ***p < 0.001); **K**–**P** Tumor growth and body weight curves in subcutaneous tumor models established with WT or *Sting*-KO KPC-1199 and PDAC cells treated with the AGP regimen (mean ± SEM; ***p < 0.001)

Although the cGAS-STING pathway is a key player in antitumor immunity and can be activated by chemotherapeutic agents [31], its role in the AGP combination chemotherapy regimen for PDAC remains unknown. We compared the effects of cisplatin, gemcitabine, and albumin-bound paclitaxel to identify the main sources of STING activation during AGP treatment. Cisplatin emerged as the strongest inducer of STING activation as evidenced by STING phosphorylation (Figure S4A). Consistent with the previously reported activation of the STING pathway mediated by cGAS in the presence of cytosolic double-stranded DNA (dsDNA), we observed cytosolic accumulation of dsDNA in PDAC cells after 24 h of cisplatin treatment (Fig. 4A). Western blotting confirmed robust activation of the cGAS-STING pathway, as indicated by the increased expression of cGAS, STING, and downstream signaling proteins (Fig. 4B). Additionally, quantitative real-time PCR (qPCR) analysis showed significant upregulation of type I interferon genes (*Ifnα*, *Ifnβ*) and chemokine genes (*Ccl5*, *Cxcl9*, *Cxcl10*) in cisplatin-treated PDAC cells (Fig. 4C).

To further investigate the role of the cGAS-STING pathway in AGP regimen-induced antitumor activity, we generated STING knockout (*Sting*-KO) PDAC and KPC-1199 cell lines using CRISPR/Cas9 technology, and knockout efficacy was confirmed via western blotting (Fig. 4D). In *Sting*-KO cells, cisplatin-induced upregulation of type I interferon genes was significantly attenuated (Fig. 4E). Similarly, upregulation of MHC-I-related genes (*β2m*, *Tap1*, *Erap1*) was diminished in *Sting*-KO cells (Fig. 4F). Flow cytometry corroborated these findings, showing reduced MHC-I expression on *Sting*-KO cells compared with that of wild-type (WT) cells after cisplatin treatment (Fig. 4G, H). Notably, cisplatin significantly upregulated the expression of PD-L1, a known interferon-stimulated gene, at both mRNA and protein levels, as confirmed by qPCR, flow cytometry, and western blotting (Figure S4E-G). To further mimic tumor-immune cell interactions, we set up a co-culture system consisting of PDAC-OVA cells, BMDCs, and B3Z T cells. This system recapitulates tumor antigen acquisition by dendritic cells (DCs), followed by processing and presentation to T cells, finally leading to T cell activation. Cisplatin treatment significantly enhanced *Il2* promoter-driven LacZ activity in B3Z cells and led to higher IL-2 secretion in the supernatant (Fig. 4I, J). These findings indicate increased T-cell activation resulting from tumor antigen presentation, which was facilitated by cisplatin treatment of tumor cells.

Moreover, in subcutaneous tumor models established using WT, *Sting*-KO KPC-1199, and PDAC cells, the therapeutic efficacy of the AGP regimen was significantly reduced in *Sting*-KO tumors. Tumor growth curves showed that *Sting* knockout abrogated the tumor-inhibitory effects of the AGP regimen (Fig. 4K–P, Figure S4B, C). Tumor tissues from *Sting*-KO models revealed no significant increase in CD8^+^ T cells or TAMs infiltration following AGP treatment, compared to that of the WT models (Figure S4D). Altogether, cisplatin activates the cGAS-STING pathway in pancreatic cancer cells and enhances antitumor immunity. Additionally, the therapeutic efficacy of the AGP regimen is highly dependent on intact STING signaling, emphasizing the critical role of tumor-intrinsic STING expression in mediating antitumor activity and the accompanying antitumor immune responses.

### Cisplatin promotes the antitumor phenotype of TAMs via activation of the cGAS-STING pathway

**Fig. 5:**
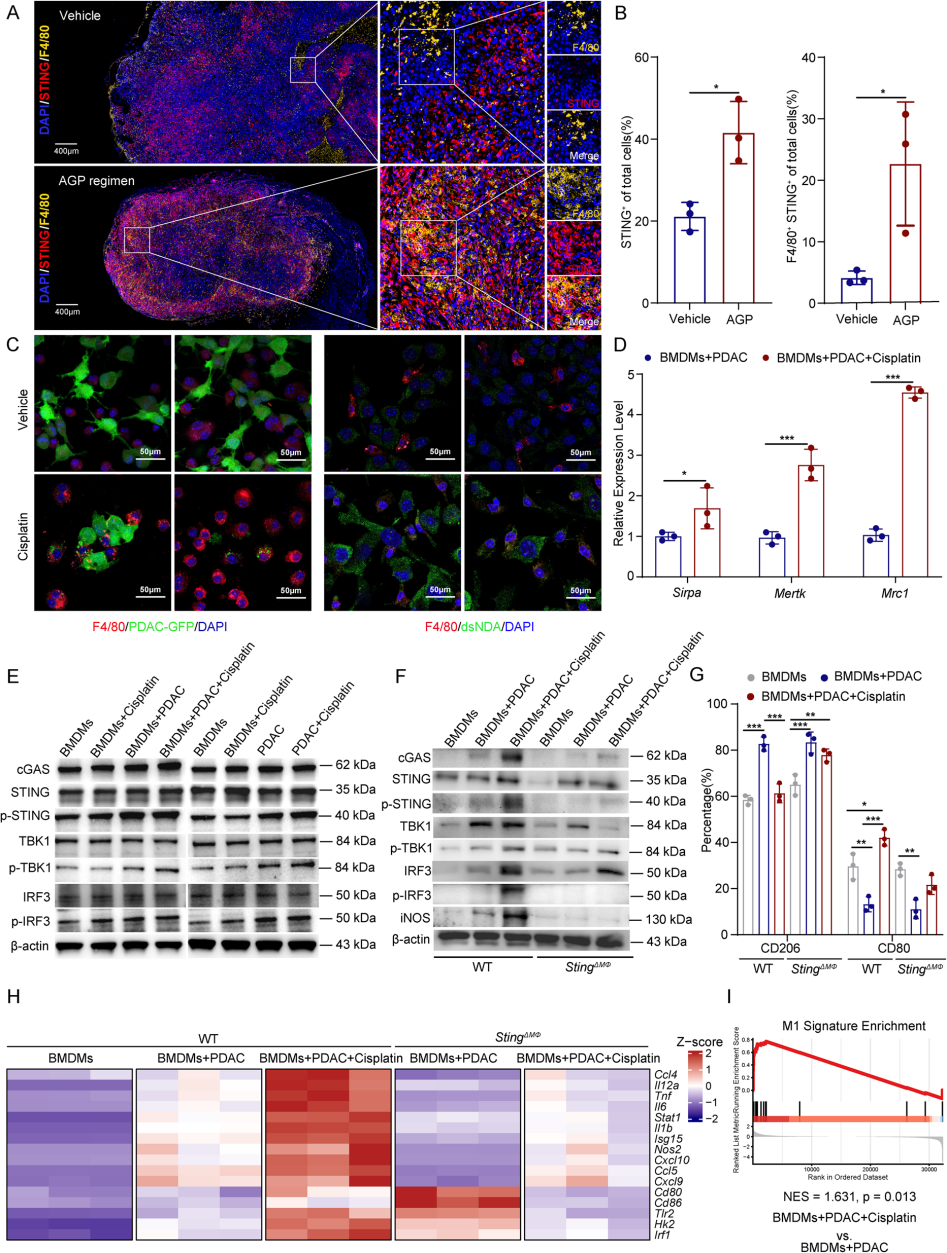
Cisplatin promotes the anti-tumor phenotype of TAMs via activation of the cGAS-STING pathway. **A**, **B** Multiplex immunofluorescence analysis of subcutaneous tumor tissues showing STING (red), TAMs (F4/80, yellow), nuclei (DAPI, blue) and co-localization (orange) (*p < 0.05); Scale bars, 400 µm; **C** Confocal fluorescence microscopy of BMDMs co-cultured with cisplatin-treated PDAC cells. Left: PDAC cells (GFP, green), BMDMs (F4/80, red), nuclei (DAPI, blue); Right: dsDNA (green); Scale bars, 50 µm; **D** qPCR analysis of phagocytosis-related gene expression (*Sirpa*, *Mertk*, *Mrc1*) in BMDMs co-cultured with PDAC cells (*p < 0.05, ***p < 0.001); **E**, **F** Western blot analysis of cGAS-STING pathway-related proteins and iNOS in BMDMs; **G** Flow cytometry analysis of CD206 (M2 marker) and CD80 (M1 marker) expression in BMDMs after cisplatin treatment (*p < 0.05, **p < 0.01, ***p < 0.001); **H**, **I** RNA sequencing analysis of a panel of M1-related genes in BMDMs

Macrophages are considered the major myeloid population essential in PDAC tumorigenesis and therapeutic response [10, 32]. We further explored the underlying mechanisms whereby AGP treatment increases the number of M1 TAMs within pancreatic tumors. Multiplex immunofluorescence analysis revealed significant activation of STING in tumor tissues following AGP treatment, with a marked increase in TAMs infiltration, predominantly at the tumor periphery. Co-localization analysis confirmed that STING expression in TAMs was significantly elevated following AGP treatment (Fig. 5A, B). In contrast, multiplex immunofluorescence demonstrated low baseline STING expression in the TAMs of human PDAC samples (Figure S5A, B).

To elucidate the mechanism underlying TAM reprogramming, we isolated BMDMs and co-cultured them with PDAC cells. Confocal microscopy revealed that cisplatin treatment markedly enhanced the phagocytic capacity of BMDMs, particularly toward damaged PDAC cells. Notably, dsDNA accumulation was observed in the BMDMs, likely originating from the phagocytosis of tumor-derived dsDNA (Fig. 5C). qPCR analysis revealed a significant upregulation of phagocytosis-associated genes (*Sirpa*, *Mertk*, and *Mrc1*) in BMDMs after cisplatin treatment (Fig. 5D). Notably, flow cytometry revealed that cisplatin exerted stronger cytotoxicity against PDAC cells than against BMDMs in co-culture (Figure S5C). Additionally, western blotting demonstrated robust activation of the cGAS-STING pathway in BMDMs following co-culture with cisplatin-treated PDAC cells (Fig. 5E). In contrast, direct cisplatin treatment of BMDMs alone did not strongly activate this pathway (Fig. 5E), indicating that phagocytosis of damaged tumor cells, rather than cisplatin-induced intrinsic DNA damage, was the primary source of STING activation in BMDMs. Besides, in human PDAC samples, a distinct spatial proximity exists between tumor cells and TAMs. This spatial arrangement provides a structural basis for potential intercellular interactions, particularly phagocytosis (Figure S5D).

To confirm that phagocytosed dsDNA drives cGAS-dependent sensing, we pre-treated WT BMDMs with the specific cGAS inhibitor RU.521 before co-culture, which significantly suppressed the cisplatin-induced upregulation of cGAS-STING pathway proteins. This finding was further validated using BMDMs derived from *cGas* knockout mice, where the activation response was significantly attenuated (Figure S5E). Furthermore, to establish the necessity of phagocytosis, we inhibited this process using Cytochalasin D. Pre-treatment with Cytochalasin D significantly suppressed the cisplatin-induced upregulation of cGAS-STING pathway proteins (Figure S5F).

To comprehensively characterize the role of the cGAS-STING pathway in cisplatin-induced macrophage polarization, we generated STING-deficient BMDMs derived from *Sting*^*f/f*^ Lyzs-CRE mice, and STING protein knockout was confirmed by western blotting (Figure S5G). Cisplatin-induced iNOS upregulation was significantly attenuated in STING-deficient BMDMs compared with that of WT BMDMs (Fig. 5F). Flow cytometry showed that cisplatin downregulated CD206 and upregulated CD80 expression in WT BMDMs, these effects were markedly diminished in STING-deficient BMDMs (Fig. 5G). To obtain a more definitive transcriptional profile, we performed RNA sequencing. The results demonstrated that co-culture with cisplatin-damaged tumor cells significantly upregulated a panel of M1-related genes in WT BMDMs, a response that was substantially diminished in STING-deficient BMDMs (Fig. 5H, I). Consistent with this pro-inflammatory transcriptional reprogramming, ELISA of co-culture supernatants demonstrated that cisplatin significantly increased CCL5, CXCL10, IFN-β, IL-6, and TNF-α secretion; however, these effects were substantially reduced in STING-deficient BMDMs (Figure S5H). Finally, to assess functional consequence, we established a co-culture system consisting of PDAC-OVA cells, BMDMs, and B3Z T cells. Cisplatin treatment led to a significant increase in IL-2 secretion, indicating enhanced macrophage-mediated antigen presentation and T-cell priming (Figure S5I). Collectively, cisplatin promoted the antitumor phenotype of TAMs by activating the cGAS-STING pathway. This activation required phagocytosis of cisplatin-damaged tumor cells, highlighting the critical role of cisplatin-stimulated tumor-macrophage crosstalk in modulating the TME.

### STING in TAMs is essential for the efficacy of the AGP regimen in pancreatic cancer models

**Fig. 6:**
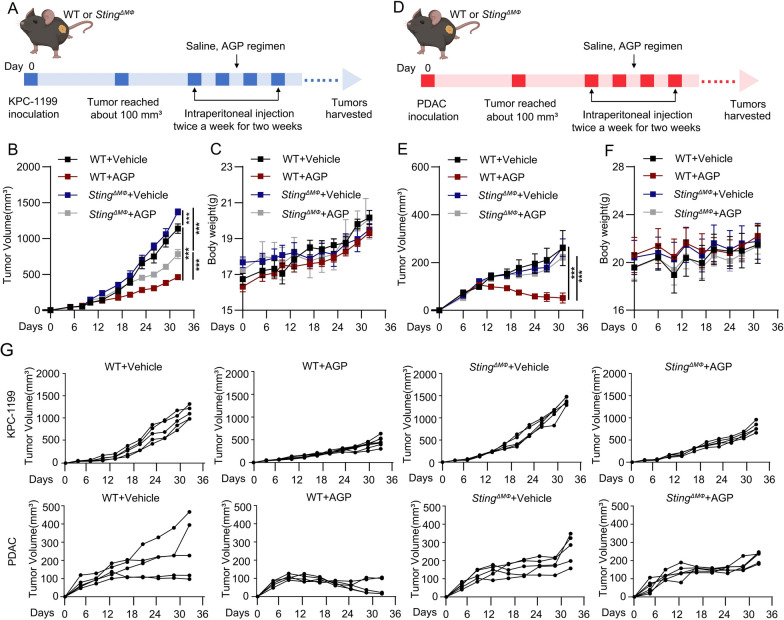
STING in TAMs is essential for the efficacy of the AGP regimen in pancreatic cancer models. **A**–**F** Tumor growth and body weight curves in subcutaneous tumor models established with KPC-1199 and PDAC cells in WT and *Sting*^*f/f*^ Lyzs-CRE mice treated with the AGP regimen (mean ± SEM; ***p < 0.001); **G** Individual tumor growth trajectories in subcutaneous models (n ≥ 5 per group)

To further investigate the role of STING in TAMs during AGP treatment, we constructed a subcutaneous pancreatic tumor model using *Sting*^*f/f*^ Lyzs-CRE mice. Notably, the therapeutic efficacy of the AGP regimen was significantly lower in the *Sting*^*f/f*^ Lyzs-CRE model than in the WT mice (Fig. 6A–G). These findings highlight the essential role of STING in TAMs for mediating the efficacy of the AGP regimen in pancreatic cancer.

### STING expression in PDAC tumor tissues correlates with an improved TIME and better prognosis

**Fig. 7:**
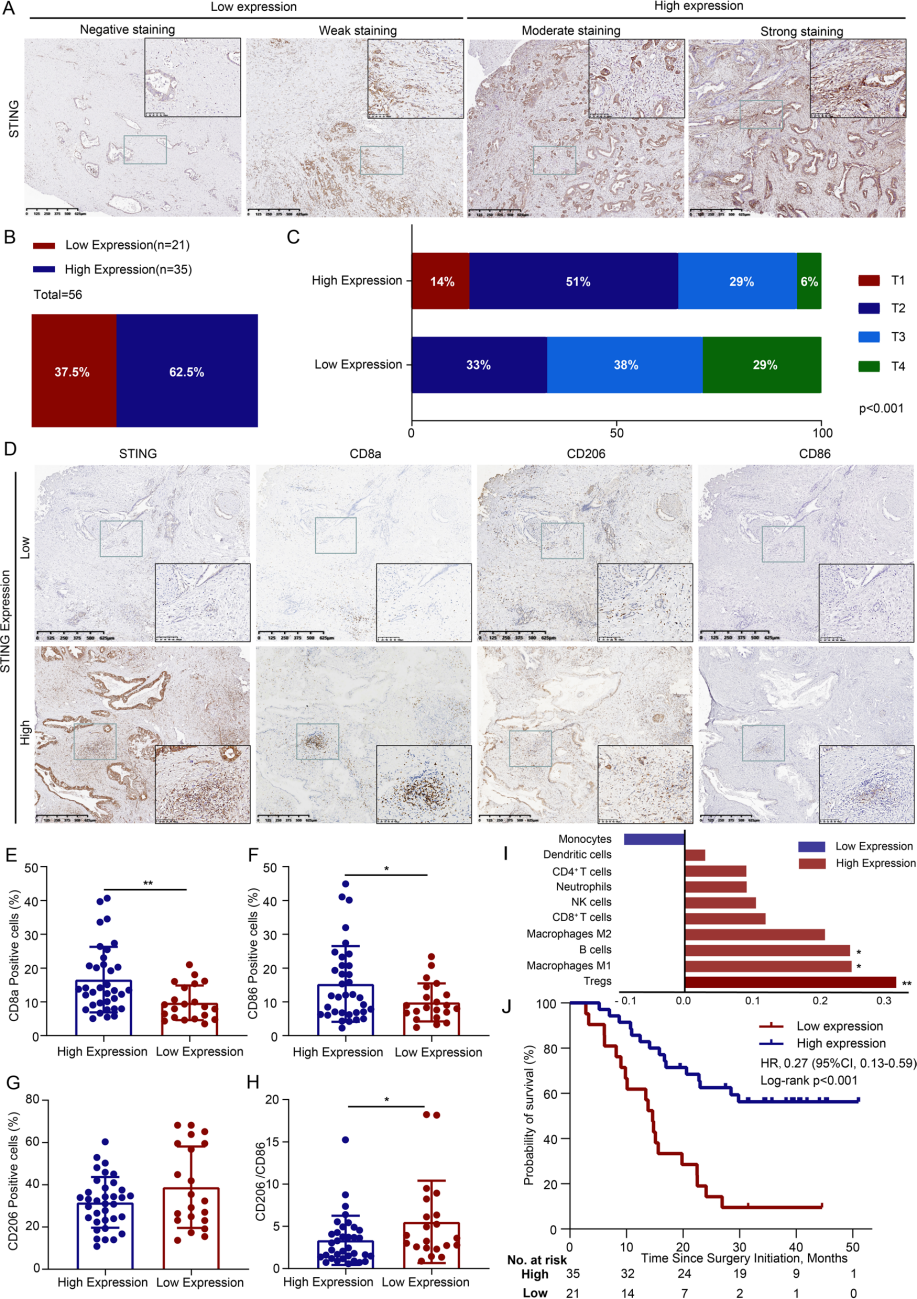
STING expression in PDAC tumor tissues correlates with an improved tumor immune microenvironment and better prognosis. **A** Representative immunohistochemistry images showing STING expression in PDAC tumor tissues;Positive signals in brown, and nuclei in blue (4 × magnification); **B** Distribution of patients according to STING expression levels; **C** Correlation between STING expression and T stage (p < 0.001); **D** IHC analysis of CD8a, CD206, and CD86 expression in PDAC tissues; Positive signals in brown, and nuclei in blue (4 × and 20 × magnification); **E**–**H** Statistical analysis of association between STING expression and infiltration of CD8a, CD86, and CD206 (*p < 0.05, **p < 0.01); **I** TCGA database analysis of the correlation between STING expression and immune cell infiltration in PDAC tissues (**p < 0.01, ***p < 0.001); **J** Kaplan–Meier survival analysis showing the relationship between STING expression and OS in PDAC patients (HR = 0.27, 95% CI 0.13–0.59, log-rank p < 0.001)

Finally, we retrospectively explored STING expression in tumor tissues from 56 patients with PDAC using immunohistochemistry. The patient characteristics are summarized in Table 4. STING expression varied among tumor samples (Fig. 7A, B) and was inversely correlated with tumor progression, as evidenced by decreased STING expression with advancing T stage (Fig. 7C).

**Table 4:** Patient and tumor characteristics

Patient characteristics	
Total patients	56
Median age, years (range)	58.32 (34–77)
Gender, male/female	32/24
Tumor site, uncus or head/body or tail	39/17
Median tumor size, cm (range)	3.75 (1.4–16)
T-classification, T1/T2/T3/T4	5/25/18/8
Lymphatic metastasis, N0/N1/N2	24/17/15
Nerves invasion, No/Yes	8/48
Vascular invasion, No/Yes	28/28
CA19-9 > 35U/ml, No/Yes	15/41

To evaluate the relationship between STING expression and immune cell infiltration, we performed immunohistochemistry for CD8a, CD86, and CD206 in the PDAC tissues (Fig. 7D). Statistical analysis revealed that high STING expression was associated with increased infiltration of CD8a^+^ T cells and CD86^+^ TAMs, whereas CD206^+^ TAMs were less abundant in tumors with high STING expression (Fig. 7E–H). In the TCGA database analysis, it was found that a strong positive correlation between STING expression and the infiltration of Tregs, M1 TAMs and B cells, and a negative correlation with monocytes in PDACs (Fig. 7I).

Survival analysis revealed that high STING expression was significantly associated with improved survival in patients with PDAC (Fig. 7J; HR = 0.27, 95% CI 0.13–0.59, log-rank p < 0.001). In addition, based on the results of our univariate and multivariate analyses (Table 5), STING expression was shown to be an independent prognostic factor for OS (HR = 0.250, 95% CI 0.123–0.507, p < 0.001). These analyses also showed that TNM classification (p = 0.027), and vascular invasion (p = 0.020) were significantly associated with OS. Further multivariate analysis showed that vascular invasion could also independently predict OS (HR = 2.317, 95% CI 1.155–4.647, p = 0.018). These results demonstrate that elevated STING expression in PDAC tumor tissues is associated with a favorable immune microenvironment and correlates with improved patient prognosis. These findings highlight STING as both a prognostic biomarker and potential therapeutic target for PDAC.

**Table 5:** Univariate and multivariate analyses of prognostic factors for overall survival

Characteristics	OS		
	Univariate	Multivariate	
		HR (95% CI)	*P* value
Gender			
Female	0.324	n.d	n.d
Male			
Age			
≤ 65	0.085	n.d	n.d
> 65			
Nerves invasion			
No	0.196	n.d	n.d
Yes			
Tumor site			
Head/Neck	0.176	n.d	n.d
Body/Tail			
STING			
Low	< 0.001*	Reference	< 0.001*
High		0.250 (0.123—0.507)	
TNM classification			
I + II	0.027*		Not significant
III			
Vascular invasion			
No	0.020*	Reference	0.018*
Yes		2.317 (1.155—4.647)	
CA19-9			
≤ 35U/ml	0.972	n.d	n.d
> 35U/ml			

*OS* overall survial, *HR* Hazard ratios, *95% CI* the corresponding 95% confidence intervals, *CA19-9* carbohydrate antigen 19-9, *TNM* tumor node metastasis, *n.d.* not done

^*^indicates statistical significance

## Discussion

**Fig. 8:**
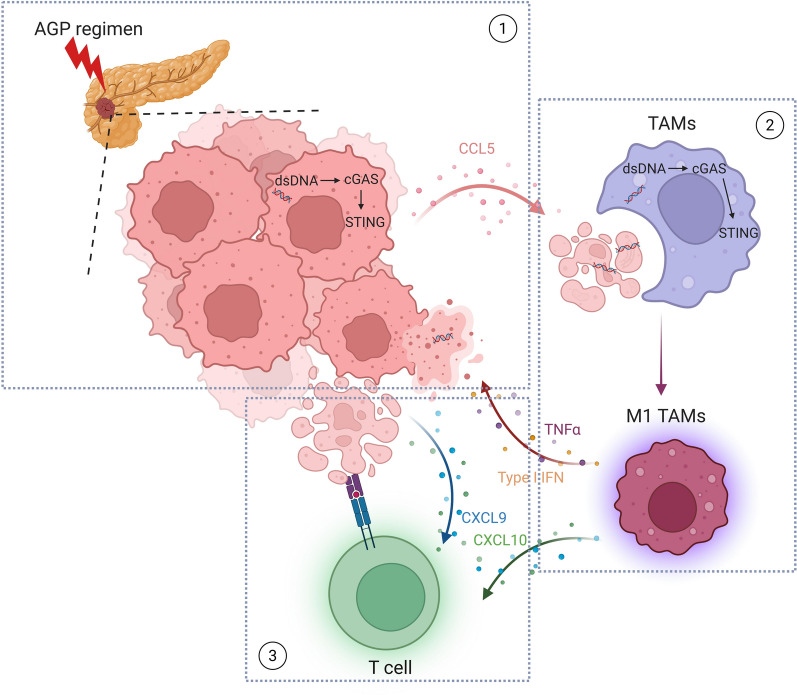
Graphical Abstract. In pancreatic cancer tumor tissues, after receiving the AGP regimen treatment, on the one hand, the cGAS-STING pathway in pancreatic cancer cells is activated, upregulating the expression of MHC-I and PD-L1, and secreting type I interferon and chemokines such as CXCL9 and CXCL10 to recruit and activate CD8^+^ T cells, initiating antitumor immunity. On the other hand, the secretion of chemokines such as CCL5 can recruit TAMs. TAMs activate the cGAS-STING pathway by phagocytosing damaged tumor cells and their released dsDNA, promoting the M1 antitumor phenotype, further improving the tumor immune microenvironment, thereby increasing the efficacy against pancreatic cancer.

Our study revealed that the AGP regimen critically enhanced PDAC treatment efficacy by reprogramming the TIME through a cGAS-STING signaling relay from tumor cells to TAMs. This dual-phase activation is initiated by cisplatin-induced DNA damage in tumor cells and amplified through tumor engulfment by TAMs. These findings provide mechanistic insights into how the AGP regimen overcomes the immunosuppressive defenses of PDAC (Fig. 8).

In our clinical cohort, the AGP regimen was associated with a significant improvement in PFS but not in OS. This discrepancy is not uncommon in oncology trials, particularly those involving aggressive malignancies and limited sample sizes [33, 34]. It may be influenced by several factors, including the receipt of effective subsequent therapies after progression, which can modulate OS independently of first-line treatment effect [35]. Furthermore, this translational study primarily aimed to elucidate the mechanism by which cisplatin augments the AG regimen; the PFS benefit provides a clinically relevant correlate to the potent immunomodulatory and anti-tumor activity demonstrated in our preclinical models. The hypothesis-generating clinical data on efficacy, combined with the robust mechanistic insights, strongly support the need for larger, prospective trials to determine whether this PFS advantage can translate into a definitive OS benefit.

In the study of mechanisms, all three agents in the AGP regimen activated the cGAS-STING pathway in PDAC cells, with cisplatin exhibiting the most potent effect. This positions cisplatin as the cornerstone of the AGP regimen, driving the robust activation of the cGAS-STING pathway. Although nab-paclitaxel enhances the cGAS-STING pathway in triple-negative breast cancer [36] and gemcitabine may impair CD8^+^ T cell function [37], the combination in the AGP regimen synergistically amplifies pathway activation and markedly upregulates type I interferon genes and MHC-I expression. This cascade recruits and activates CD8^+^ T cells, leading to potent antitumor immunity. Mechanistically, cisplatin-damaged PDAC cells release damage-associated molecular patterns (DAMPs), such as dsDNA, which are engulfed by TAMs, triggering secondary cGAS-STING pathway activation. This reprograms TAMs toward an M1 antitumor phenotype, in contrast to that of STING agonist strategies in breast cancer [38]. We propose a novel “STING relay” model: cisplatin initiates signaling in tumor cells, which is propagated to TAMs via DAMP transfer, converting immunologically “cold” tumors to “hot” microenvironments. This dual-phase activation underscores the critical role of tumor-immune crosstalk in reshaping the TIME and enhancing therapeutic efficacy.

Our findings further established that activation of the cGAS-STING pathway in TAMs is dependent on the phagocytosis of cisplatin-damaged PDAC cells. This mechanism was validated through direct co-culture assays and was consistent with previous findings in a melanoma model [39]. Cisplatin alone failed to activate STING in TAMs in the absence of tumor cell debris, highlighting the necessity of tumor-derived DAMPs as mediators of innate immune activation. The AGP regimen leverages this phagocytosis-driven “STING relay” to orchestrate multidimensional immune modulation: cisplatin induces primary STING activation in tumor cells via DNA damage, whereas TAMs that engulf cisplatin-induced tumor cell debris undergo secondary STING signaling, polarizing them to an M1 phenotype. This reprogramming is further supported by the unique ability of albumin-bound paclitaxel to enhance macrophage uptake of immunogenic cargo [40], collectively counteracting gemcitabine resistance driven by M2 TAMs [41]. Spatial immunofluorescence analyses revealed that the AGP regimen redistributed TAMs to the tumor periphery, a STING-dependent process mediated by chemokines and debris from cisplatin-treated PDAC cells, mirroring the myeloid cell spatial dynamics observed in other malignancies [42]. This reorganization establishes a pro-inflammatory frontier at tumor edge to facilitate CD8^+^ T-cell infiltration and immune surveillance. Previous strategies targeting TAMs, such as macrophage depletion [43] or CD40 agonist monotherapy [44], have shown limited clinical efficacy owing to their impracticality or lack of survival benefit. However, the AGP regimen redefines TAM engagement by functionally reprogramming rather than eliminating or directly activating these cells, thereby preserving their immunostimulatory potential.

Prognostically, high STING expression in PDAC correlates with improved survival and an immune-active microenvironment, a pattern also observed in non-small cell lung cancer (NSCLC), hepatocellular carcinoma, and gastric cancer [45–47], with the co-expression of cGAS and STING further refining prognostic stratification [48]. These findings suggest that STING is a valuable biomarker for predicting the outcomes of patients with PDAC. Notably, the AGP regimen-induced adaptive PD-L1 upregulation in tumor and myeloid cells aligns with the mechanisms observed in NSCLC, where platinum-based chemotherapy synergizes with PD-1/PD-L1 inhibitors [49, 50]. This observation raises the question of whether adding PD-1/PD-L1 inhibitors to the AGP regimen could further enhance efficacy. Preclinical evaluation of this combination is warranted and, if synergistic, could represent a promising strategy to overcome immunotherapy resistance in PDAC. In summary, the AGP regimen reimagines PDAC therapy by bridging cytotoxic and immunotherapeutic principles through STING-dependent TAM reprogramming, spatial microenvironment remodeling, and biomarker-guided precision, thereby offering a transformative paradigm for this intractable malignancy.

This study has certain limitations. First, the clinical data analysis was retrospective, single-center, and involved a relatively small sample size, which may have introduced selection bias. Prospective multicenter studies with larger cohorts are required to validate our results. Second, although we demonstrated the importance of the cGAS-STING pathway in the AGP regimen, the precise molecular interactions between the tumor and immune cells require further investigation. Advanced techniques, such as single-cell RNA sequencing and spatial transcriptomics, can provide deeper insights into the dynamic changes within the TME. Third, future studies comparing pre- and post-AGP treatment tissue samples could further dissect the remodeling of the immune microenvironment induced by this regimen. Finally, the potential synergistic effects of AGP and immune checkpoint inhibitors warrant further investigation in preclinical and clinical trials.

## Conclusion

This study established that the AGP regimen is a promising potential therapeutic strategy for PDAC, with cisplatin-mediated activation of the cGAS-STING pathway playing a pivotal in its efficacy. By triggering a previously unrecognized cGAS-STING signaling relay between tumor cells and TAMs, the AGP regimen reshapes the TIME and enhances antitumor immunity, thereby providing a new approach to overcoming the challenges of treating this immunologically cold tumor. These findings provide a robust groundwork for future PDAC therapies aimed at optimizing chemotherapy regimens, identifying predictive biomarkers, and exploring synergistic combinations with immunotherapy.

## Supplementary Information

Supplementary material 1.

## Data Availability

The data that support the findings of this study are available from the corresponding author upon reasonable request.

## Abbreviations

PDAC: Pancreatic ductal adenocarcinoma
APC: Advanced pancreatic cancer
AG regimen: Gemcitabine plus albumin-bound paclitaxel
AGP regimen: AG plus cisplatin
DAMPs: Damage-associated molecular patterns
TME: Tumor microenvironment
TIME: Tumor immune microenvironment
Tregs: Regulatory T cells
MDSCs: Myeloid-derived suppressor cells
TAMs: Tumor-associated macrophages
TANs: Tumor-associated neutrophils
BMDMs: Bone marrow-derived macrophages
DCs: Dendritic cells
SYSUCC: Sun Yat-sen University Cancer Center
ECOG: Eastern Cooperative Oncology Group
FBS: Fetal bovine serum
PBS: Phosphate-buffered saline
ELISA: Enzyme-linked immunosorbent assay
CyTOF: Mass cytometry
ORR: Objective response rate
CI: Confidence intervals
HR: Hazard ratio
PR: Partial response
SD: Stable disease
OS: Overall survival
PFS: Progression-free survival
WT: Wild-type
NSCLC: Non-small cell lung cancer
