# Supplementary material for: A STING signaling relay from tumor cells to macrophages mediates the improved efficacy of combination chemotherapy in pancreatic cancer
Source: J Biomed Sci. 2026 Mar 4;33:26. doi: 10.1186/s12929-026-01226-1 (PMC12961847; doi:10.1186/s12929-026-01226-1)
Supplement: Supplementary file 1 — Supplementary material 1. [file 12929_2026_1226_MOESM1_ESM.docx]

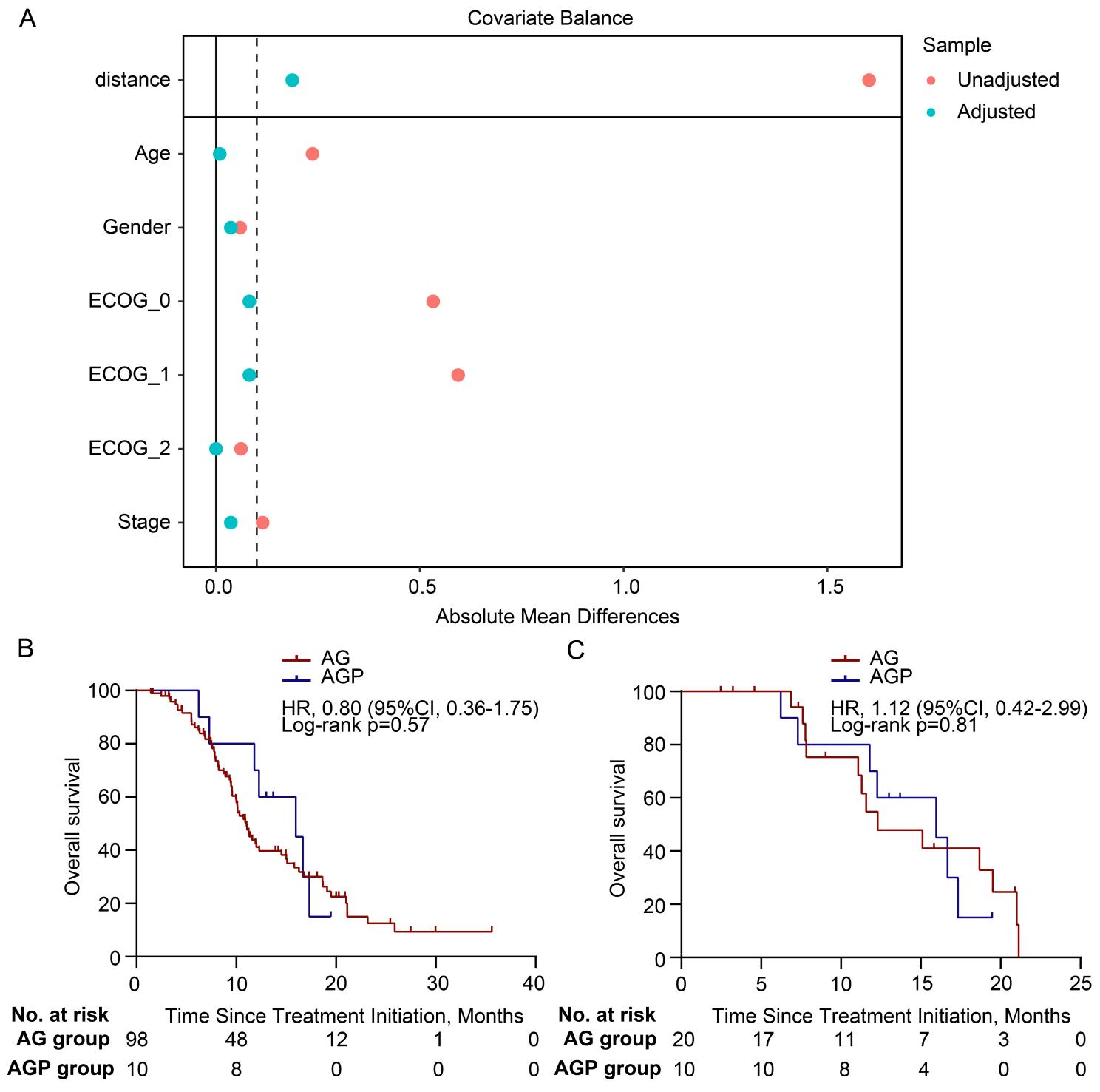


**Figure S1. Clinical response to the AGP regimen**

1. The balance diagnostics results.

B-C. Kaplan-Meier survival curves showing no significant difference of OS in the AGP regimen group compared to the AG regimen group, both in unmatched and matched cohorts.

**
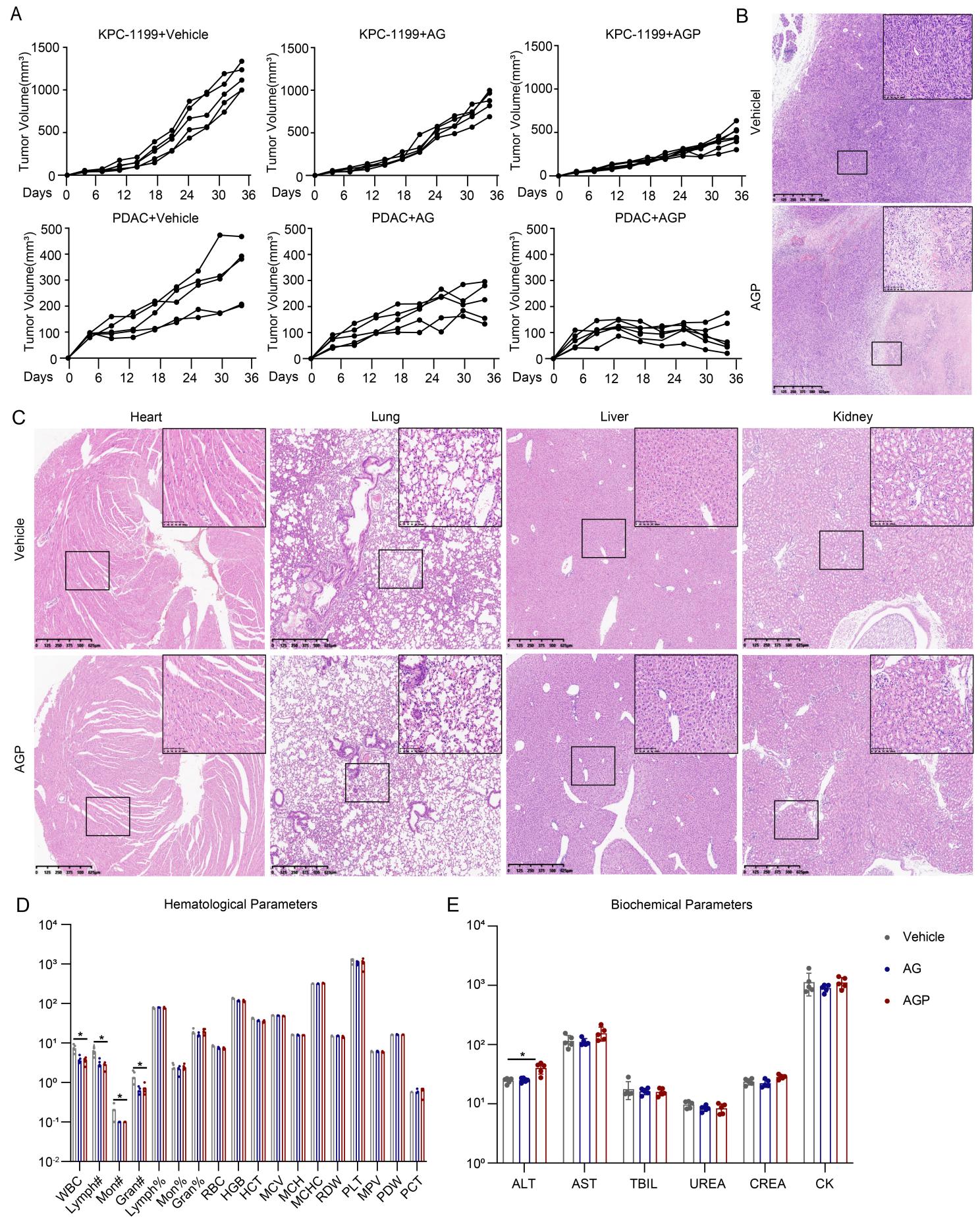
**

**Figure S2. Therapeutic efficacy of the AGP regimen in mouse pancreatic cancer models**

A. Individual tumor growth trajectories in subcutaneous models (n ≥ 5 per group).

B. H&E-stained tumor sections showing necrotic areas in AGP regimen group (4× and 20× magnification).

C. Histopathological analysis of major organs confirming treatment safety (4× and 20× magnification).

D, E. Hematology and serum biochemistry analyses (*p < 0.05).

**
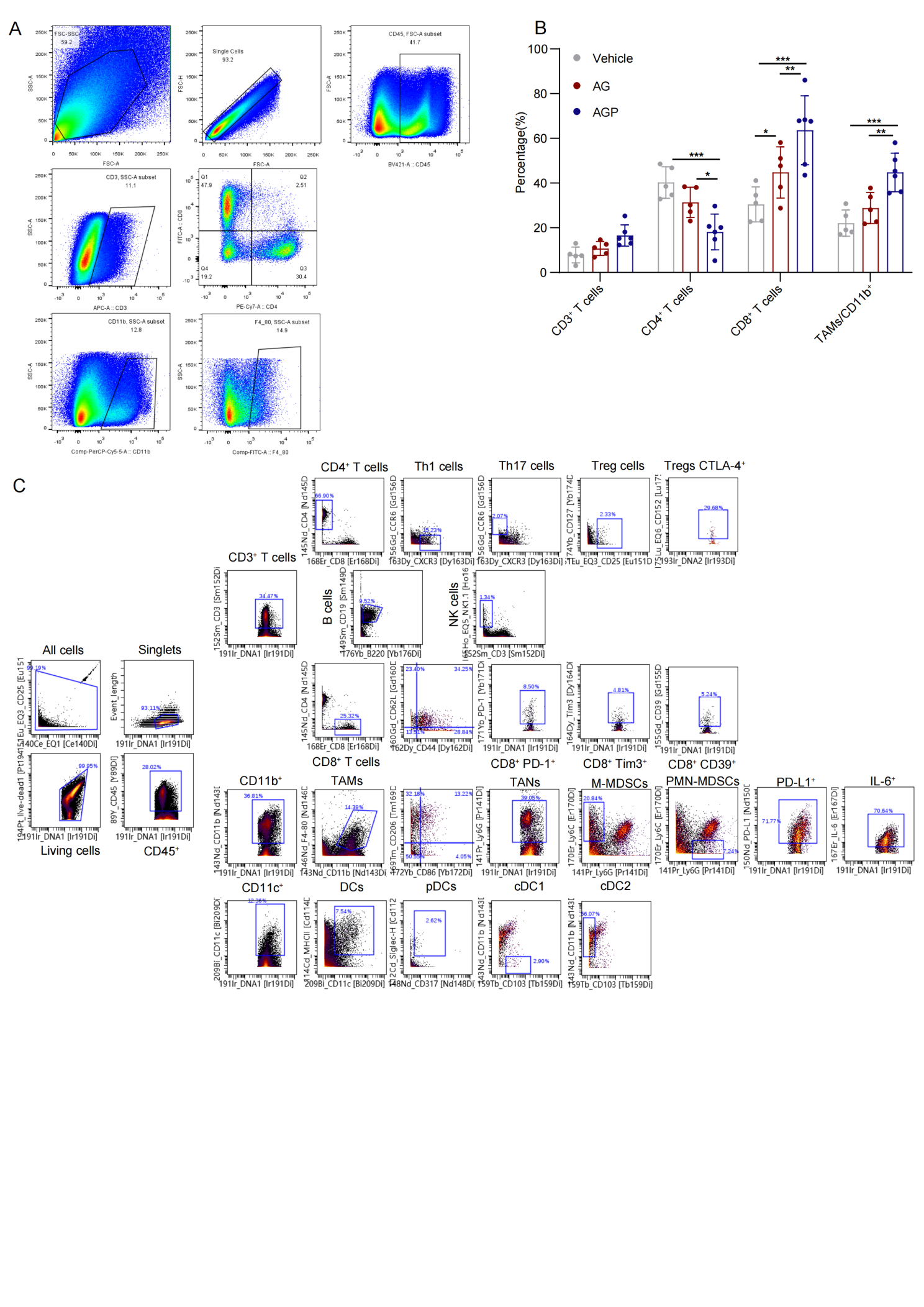
**

**Figure S3. Changes in immune markers and subsets of immune cells**

A. Gating strategy of flow cytometry.

B. Quantitative analysis of immune cell infiltration changes among the control group, AG regimen group, and AGP regimen group (*p < 0.05, ***p < 0.001).

C. Gating strategy of CyTOF.

**
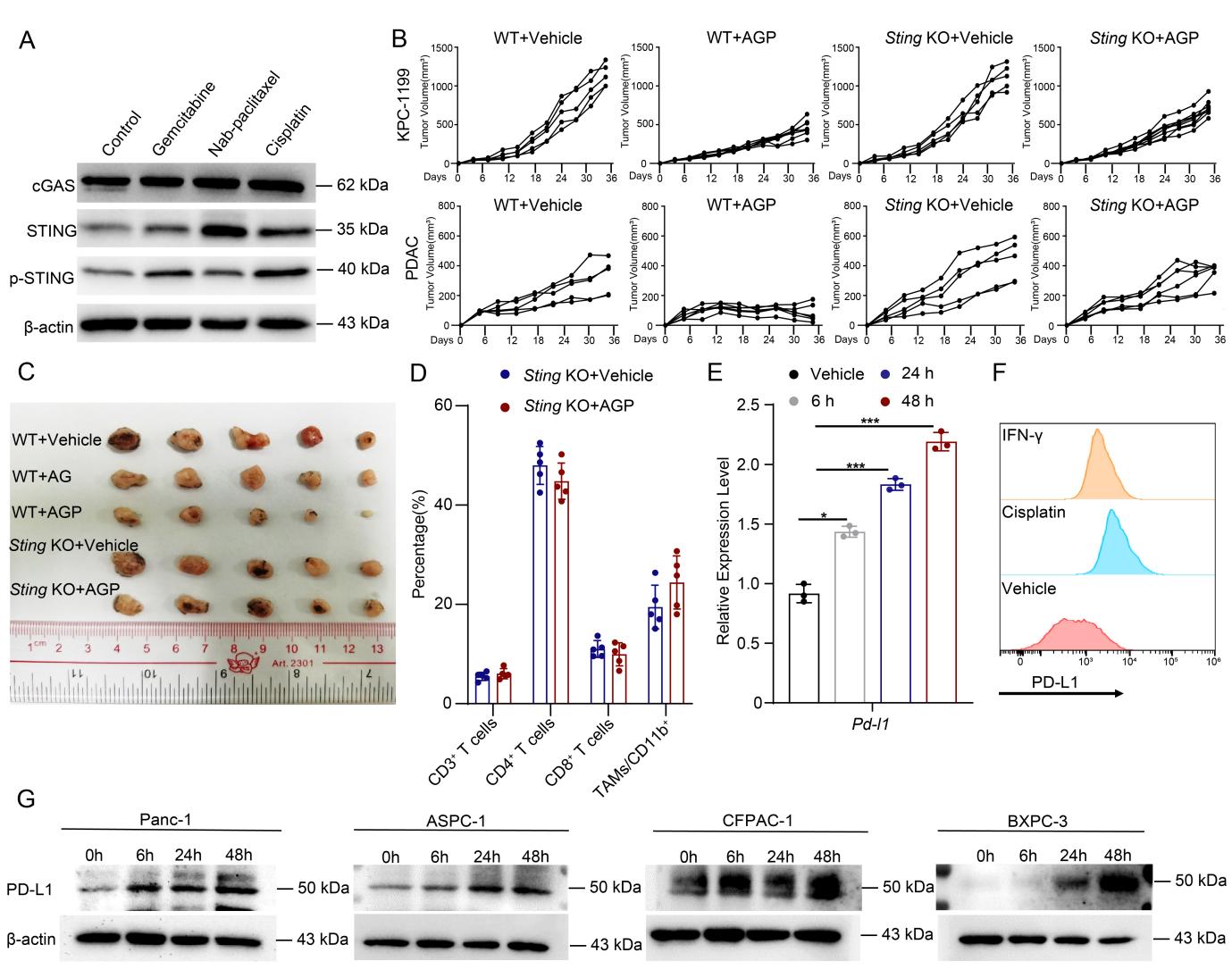
**

**Figure S4. Cisplatin activated the cGAS-STING pathway in pancreatic cancer**

A. Western blot analysis of cGAS-STING pathway activation in PDAC cells treated with different chemotherapy drugs.

B-C. Individual tumor growth curves and representative tumor images in subcutaneous models (n ≥ 5 per group).

D. Quantitative analysis of immune cell infiltration changes in tumor tissues from *Sting*-KO models (*p < 0.05, ***p < 0.001).

E-G. qPCR, flow cytometry, and western blot analysis of PD-L1 expression in cisplatin-treated PDAC cells (*p < 0.05, ***p < 0.001).

**
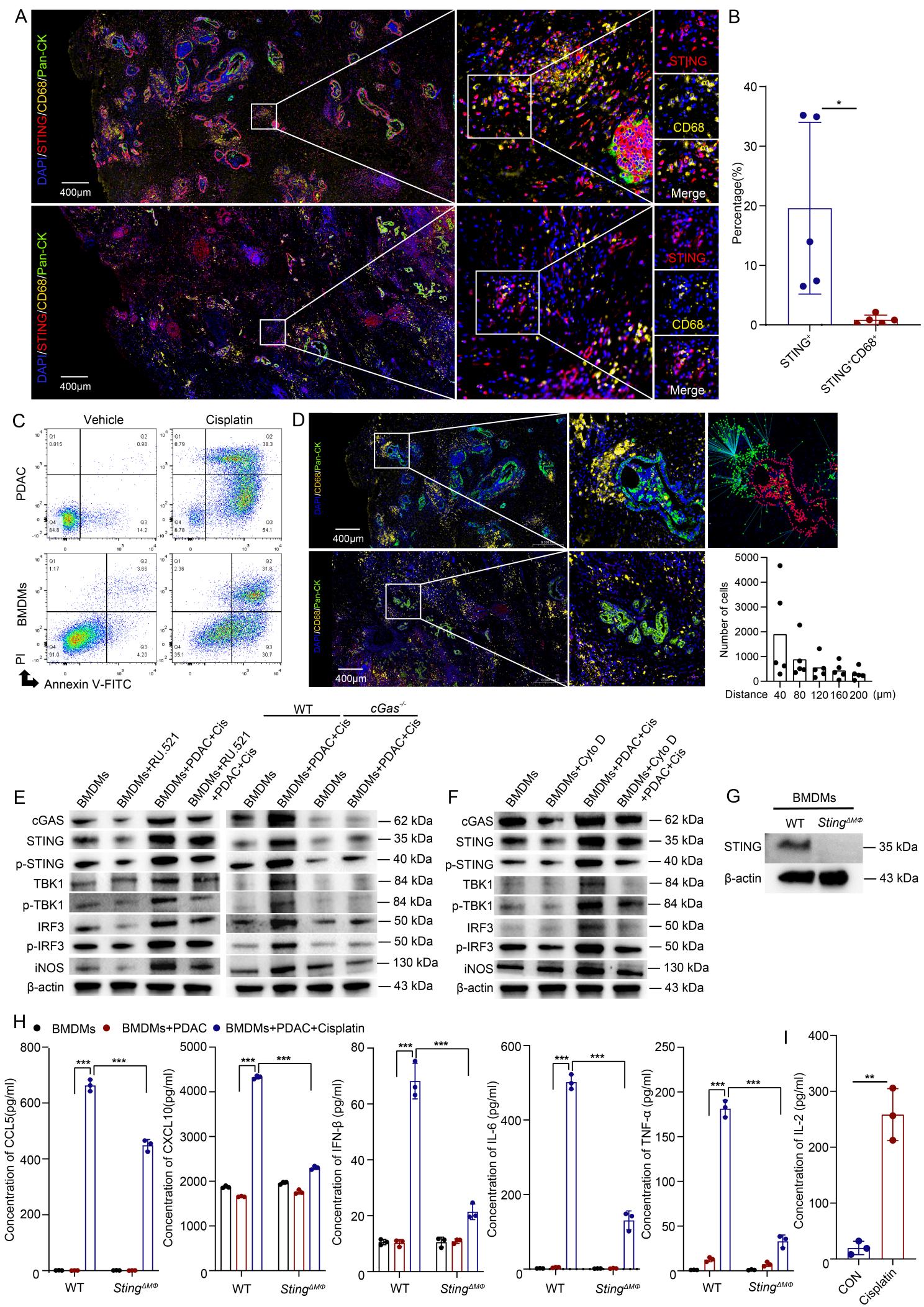
**

**Figure S5. Cisplatin induced TAMs reprogramming in PDAC.**

A-B. Multiplex immunofluorescence analysis of tumor tissues from patients with pancreatic cancer showing STING (red), TAMs (CD68, yellow), pancreatic cancer cells (Pan-CK, green), and nuclei (DAPI, blue). Scale bars, 400 µm (*p < 0.05).

1. Flow cytometry analysis of cisplatin cytotoxicity on PDAC cells and BMDMs in the co-culture system.
2. Multiplex immunofluorescence analysis of tumor tissues from patients with pancreatic cancer showing TAMs (CD68, yellow), pancreatic cancer cells (Pan-CK, green), and nuclei (DAPI, blue). Counting the number of TAMs within a certain distance around pancreatic cancer cells in tumor samples from 5 patients (Mean); Scale bars, 400 µm.

E- F. Western blot analysis of cGAS-STING pathway-related proteins and iNOS in BMDMs;

G. Western blot validation of STING knockout in BMDMs.

H-I. ELISA detection of CCL5, CXCL10, IFN-β, IL-6, and TNF-α levels in the co-culture supernatant (*p < 0.05, **p < 0.01, ***p < 0.001).

**Table S1.** **Detection markers and channels of CyTOF.**

| Markers | Channels | Markers | Channels | Markers | Channels |
| --- | --- | --- | --- | --- | --- |
| CD45 | 89Y | CD69 | 154Sm | Ly6c | 170Er |
| Siglec-H | 112Cd | CD39 | 155Gd | PD-1 | 171Yb |
| MHC II | 114Cd | CCR6 | 156Gd | CD86 | 172Yb |
| EQ1 | 140Ce | CD103 | 159Tb | Granzyme | 173Yb |
| Ly6G | 141Pr | CD62L | 160Gd | CD127 | 174Yb |
| EQ2 | 142Ce | IL-10 | 161Dy | CD152 | 175Lu |
| CD11b | 143Nd | CD44 | 162Dy | EQ7 | 176Lu |
| CD4 | 145Nd | CXCR3 | 163Dy | B220 | 177Yb |
| F4-80 | 146Nd | Tim3 | 164Dy | DNA1 | 191Ir |
| CD317 | 148Nd | NK1.1 | 165Ho | DNA2 | 193Ir |
| CD19 | 149Sm | SIRPa | 166Er | Live-dead1 | 195Pt |
| PD-L1 | 150Nd | IL-6 | 167Er | Live-dead2 | 195Pt |
| CD25 | 151Eu | CD8 | 168Er | Live-dead3 | 198Pt |
| CD3 | 152Sm | CD206 | 169Tm | CD11C | 209Bi |

**Table S2.** **Primers sequences.**

| Gene | Forward primer (5’ - 3’) | Reverse primer (5’ - 3’) |
| --- | --- | --- |
| *β-actin* | AGAGGGAAATCGTGCGTGAC | CAATAGTGATGACCTGGCCGT |
| *Ifn-α* | TGATGAGCTACTACTGGTCAGC | GATCTCTTAGCACAAGGATGGC |
| *Ifn-β* | CAGCTCCAAGAAAGGACGAAC | GGCAGTGTAACTCTTCTGCAT |
| *Ccl5* | GCTGCTTTGCCTACCTCTCC | TCGAGTGACAAACACGACTGC |
| *Cxcl9* | CCTAGTGATAAGGAATGCACGATG | CTAGGCAGGTTTGATCTCCGTTC |
| *Cxcl10* | CCAAGTGCTGCCGTCATTTTC | GGCTCGCAGGGATGATTTCAA |
| *Pd-l1* | TGCGGACTACAAGCGAATCACG | CTCAGCTTCTGGATAACCCTCG |
| *Il-2* | GCGGCATGTTCTGGATTTGACTC | CCACCACAGTTGCTGACTCATC |
| *Sirpa* | TCATCTGCGAGGTAGCCCACAT | ACTGTTGGGTGACCTTCACGGT |
| *Mertk* | ATCATCCTCGGCTGCTTCTGTG | ACGACCAGTTGGGAATCCTCCT |
| *Mrc1* | GTTCACCTGGAGTGATGGTTCTC | AGGACATGCCAGGGTCACCTTT |
| *Inos* | GAGACAGGGAAGTCTGAAGCAC | CCAGCAGTAGTTGCTCCTCTTC |
| *Arg1* | CATTGGCTTGCGAGACGTAGAC | GCTGAAGGTCTCTTCCATCACC |
| *β2m* | TTCTGGTGCTTGTCTCACTGA | CAGTATGTTCGGCTTCCCATTC |
| *Tap1* | GGACTTGCCTTGTTCCGAGAG | GCTGCCACATAACTGATAGCGA |
| *Erap1* | TAATGGAGACTCATTCCCTTGGA | AAAGTCAGAGTGCTGAGGTTTG |
